# Secondary (iso)BAs cooperate with endogenous ligands to activate FXR under physiological and pathological conditions

**DOI:** 10.1016/j.bbadis.2021.166153

**Published:** 2021-04-22

**Authors:** Alex Zaufel, Sandra M.W. van de Wiel, Lu Yin, Günter Fauler, Daphne Chien, Xinzhong Dong, John F. Gilmer, Jennifer K. Truong, Paul A. Dawson, Stan F. J. van de Graaf, Peter Fickert, Tarek Moustafa

**Affiliations:** a Division of Gastroenterology and Hepatology, Department of Internal Medicine, Medical University of Graz, Austria; b Tytgat Institute for Liver and Intestinal Research, & Department of Gastroenterology and Hepatology, Amsterdam University Medical Centers, University of Amsterdam, the Netherlands; c Amsterdam Gastroenterology, Endocrinology, Metabolism (AGEM), Amsterdam University Medical Centers, the Netherlands; d The School of Pharmacy and Pharmaceutical Sciences, Trinity College Dublin, Ireland; e Clinical Institute of Medical and Chemical Laboratory Diagnostics Medical, University of Graz, Austria; f Solomon H. Snyder Department of Neuroscience, Johns Hopkins University School of Medicine, Baltimore, MD 21205, United States of America; g Division of Pediatric Gastroenterology, Hepatology & Nutrition, Emory University School of Medicine, Atlanta, GA, USA

**Keywords:** Nuclear receptors, Bile acids, Enterohepatic, cholestasis, gene expression, Metabolism

## Abstract

IsoBAs, stereoisomers of primary and secondary BAs, are found in feces and plasma of human individuals. BA signaling via the nuclear receptor FXR is crucial for regulation of hepatic and intestinal physiology/pathophysiology. Aim: Investigate the ability of BA-stereoisomers to bind and modulate FXR under physiological/pathological conditions. Methods: Expression-profiling, luciferase-assays, fluorescence-based coactivator-association assays, administration of (iso)-BAs to WT and cholestatic mice. Results: Compared to CDCA/isoCDCA, administration of DCA/isoDCA, UDCA/isoUDCA only slightly increased mRNA expression of FXR target genes; the induction was more evident looking at pre-mRNAs. Notably, almost 50% of isoBAs were metabolized to 3-oxo-BAs within 4 h in cell-based assays, making it difficult to study their actions. FRET-based real-time monitoring of FXR activity revealed that isoCDCA*>*CDCA stimulated FXR, and isoDCA and isoUDCA allowed fully activated FXR to be re-stimulated by a second dose of GW4064. *In vivo* co-administration of a single dose of isoBAs followed by GW4064 cooperatively activated FXR, as did feeding of UDCA in a background of endogenous FXR ligands. However, in animals with biliary obstruction and concomitant loss of intestinal BAs, UDCA was unable to increase intestinal Fgf15. In contrast, mice with an impaired enterohepatic circulation of BAs (Asbt−/−, Ostα−/−), administration of UDCA was still able to induce ileal Fgf15 and repress hepatic BA-synthesis, arguing that UDCA is only effective in the presence of endogenous FXR ligands. Conclusion: Secondary (iso) BAs cooperatively activate FXR in the presence of endogenous BAs, which is important to consider in diseases linked to disturbances in BA enterohepatic cycling.

## 1. Introduction

The enterohepatic circulation of bile acids (BAs) from the liver to the intestine and back summarizes their primary sites of action. Primary BAs are synthesized from cholesterol in the liver and bio-transformed in the distal small intestine and colon by the gut microbiota, forming a variety of secondary BAs. In the intestine, BAs control lipid absorption and influence microbiota diversity. Given the abundance of various secondary BAs, it is remarkable how little is known about their biological importance and molecular function compared to primary BAs [1,2].

Intriguingly, microbiota research has evoked a zestful renaissance of especially secondary BAs and attracted much attention to BA-research as the gut microbiota clearly affects host health and diseases. Microbial synthesis and metabolism of BA-species with unique amino acid conjugations has been discovered recently. These BAs are found in humans, and were enriched in patients with inflammatory bowel disease (IBD) [3]. 7α-dehydroxylating bacteria promote hepatocellular carcinoma (HCC) by deoxycholic acid (DCA) synthesis, linking cancer to microbiota-derived BA metabolism [4]. In contrast, 7β-BAs such as ursodeoxycholic acid (UDCA) provide protective effects against HCC and colon cancer [4,5]. Microbial metabolism produces secondary BAs with 3-OH group in alpha configuration such as DCA, UDCA or lithocholic acid (LCA), and additionally a variety of keto-intermediates and stereoisomers with C-3 hydroxylation in beta configuration, termed isoBAs [6]. Therefore, the composition of BAs returning from the intestine to the liver is a mixture of free and conjugated, secondary, ketoand isoBAs.

Bacterial transformation of CDCA, DCA, LCA and UDCA into their corresponding isoBAs has already been described in the late ‘80s [7–9]. IsoBAs are found in feces, serum and urine reaching concentrations of up to ~200 μM in caecal contents of healthy human individuals [1]. IsoBAs have lower detergent activity and toxicity on bacteria [10], and human hepatoblastoma cell lines [11]. IsoBAs are presumably less efficient than natural BAs in solubilizing dietary lipids [12] and are less efficacious secretagogues [13]. A detailed characterization of a novel bacterial biosynthetic pathway of *e.g.* isoDCA has been described [10] with subsequent exciting work identifying a critical role of certain microbiota and isoBAs, in particular isoDCA and isoalloLCA, in immune cells. Both isoBAs affect regulatory T cells function and thereby modulate the immunological balance in the colon. [14–16].

BA can activate nuclear hormone receptors, such as the farnesoid X receptor (FXR), thereby precisely controlling BA-synthesis, transport and metabolism. Various mouse models have been used to elucidate molecular mechanisms behind FXR activation, leading to the identification of endogenous FXR-agonistic, and more recently, FXR-antagonistic BAs [17,18]. *Hsd3b7* encodes the enzyme, catalyzing the initial reaction required for the conversion of cholesterol to BAs (3β-OH group of cholesterol to the 3α-OH). In this context, 3α-hydroxylated BAs are drastically reduced in *Hsd3b7* knockout mice, but large quantities of isoBAs are produced [19]. The loss of feedback regulation of BA-synthesis in *Hsd3b7*-KO mice suggests that isoBAs interfere with FXR action. It has been speculated that isoBAs may act as FXR-antagonists, or that they compete with the uptake of normal BAs, thereby preventing FXR activation. In line, genetic deletion of the main BA-transporter in the intestine (*Asbt* knockout mice) results in a similar expression profile with almost absent FXR target genes in the intestine (*Fgf*15 expression) and drastically elevated expression levels of *Cyp*7a1 mRNA in the liver [20,21]. The same defective regulation of FXR target genes in liver and intestine is observed in animals with common bile duct ligation (CBDL), a model of obstructive cholestasis. Cholestasis literally means a standstill of bile (chole = “bile” and stasis = “standstill”). A plethora of diseases leads to cholestasis *via* different pathophysiological mechanism. Cholestasis itself has broad clinical implications and consequences since it affects multiple organs because of spillage of excess amounts of BAs into the blood stream. Importantly, luminal BAs are absent in the intestine resulting in dysbiosis and dysregulated molecular pathways in enterocytes [6].

To gain further insights into the regulatory functions of isoBAs with regard to FXR activity, we assessed the regulation of FXR by isoBAs in comparison to their 3alpha-stereoisomers *in vitro* using different cell-based assays, and *in vivo* using healthy mice and animal models with genetically or mechanical impairment of the enterohepatic circulation of BAs. We show that different isoBAs have unique properties as FXR ligands, ranging from agonists, to partial agonists or allosteric modulators. FXR-mediated target gene transcription seems to be dependent on a burst induction or long half-life of the target gene mRNA. Our data further suggest that especially secondary BAs (*e.g.* UDCA and isoUDCA) act with endogenous 3α-epimers in a unique fashion to cooperatively activate FXR. Administration of UDCA strongly increased *Fgf*15 in wild type, but not in *Fxr* knockout or CBDL-animals, the latter lacking BAs in the intestine. Similarly, UDCA rescued the very low levels of intestinal *Fgf*15 and even lowered the very high levels of hepatic *Cyp*7a1 in *Asbt* knockout mice, another genetic model of disturbed enterohepatic circulation of endogenous BAs. Notably, a combined deletion of OST*α* together with ASBT, balanced concentrations of BAs in DKO enterocytes and allowed UDCA to re-stimulate FXR and induce *Fgf15*, similar to treatment in WT animals. These selective properties of secondary (iso) BAs may facilitate a unique gene regulatory response by FXR as long as endogenous BAs are present in sufficient amounts in appropriate tissues.

## 2. Materials and methods

### Design and synthesis of isoBAs

We designed a synthetic route via 3-OH oxidation and stereoselective reduction of the resultant 3-keto compounds to obtain the iso-Bas used in this study. (Scheme 1, Suppl. material). As indicated in Scheme 1 (cholic acid is shown as an example of the general approach), the synthetic route involved addition of acetyl groups to all the hydroxyl groups except at position C-3’, while converting the 3α-hydroxyl into a 3-keto with pyridinium chlorochromate (PCC). The 3β-hydroxyl was then obtained via reduction of the keto group. This was accomplished with good stereo specificity (equatorial approach) using the bulky reducing agent L-selectride. Finally, the acetate protection was removed by hydrolysis to regenerate the hydroxyl groups. Synthesis of the 3β-OH isomers of five different bile acids (DCA, CDCA, LCA, UDCA and CA) was performed in this fashion. LCA, a monohydroxy BA with a 3α-hydroxy group, did not require the protection/deprotection steps. Compound purity and identity was confirmed using a combination of ^1^H-NMR, ^13^C-NMR, TLC and HR-MS. A detailed description of the synthetic approach adopted is in supplemental material.

### Cell culture

All cells used in this study were maintained in DMEM (4.5 g/L glucose) supplemented with 10% fetal bovine serum (FBS), 1% pen/strep and 1% l-glutamine. NucleoBAS (Bile Acid Sensor localized in the nucleus [22]) expressing U2OS cells were engineered by transfecting cells using polyethylenimine (PEI). Stable cell lines were generated by colony selection using cloning cylinders over well separated colonies as previously described [23]. Stably expressing NTCP-mKate2 cells on U2OS- NucleoBAS background were obtained by lentiviral transduction as previously described [22]. All cells were cultured at 5% CO_2_ at 37 °C.

### Cell Viability Assay

Caco-2 cells were incubated in medium containing 100 μM bile acids for 24 h. Cells were then treated with 10 μL of 2.5 mg/mL (3-(4, 5-dimethylthiazol-2-yl)-2, 5-diphenyltetrazolium bromide) MTT reagent and cultured for 2 h according to manufactures protocol. The assay was normalized to 1% DMSO vehicle control and absorbance of each well was read on a VERSAmax Microplate Reader (Molecular Devices, Sunnyvale, CA, USA) at a wavelength of 570 nm.

### Confocal microscopy based FRET-Bile Acid Sensor

When indicated in the figure legends, NucleoBAS expressing U2OS cells were cultured in medium containing 5% charcoal stripped fetal bovine serum for 7 days prior to the experiment. For live-cell confocal microscopy, cells stably expressing NucleoBAS were seeded in an 8-well coverslip bottomed chamber slide (WVR, Amsterdam, Netherlands) in Leibovitz’s L15 medium. Live cell fluorescent imaging was acquired on a Leica SP8X-SMD confocal microscope with a 63× oil objective, fully enclosed with 37 °C incubation chamber [22,23].

## 3. MRGPRX4 activity EC50 determinations

HEK293 cells stably expressing Gα15 and MRGPRX4 were seeded in poly-d-lysine–coated 96-well plates at 25,000 cells per well and incubated overnight. Cells were loaded with FLIPR Calcium 5 dye (Molecular Devices) diluted in Hanks’ balanced salt solution (HBSS) supplemented with 20 mM Hepes, pH 7.4 as described previously [24]. BAs and isoBAs were freshly dissolved and diluted into HBSS with 20 mM Hepes. Compounds were added and cells were imaged with a Flexstation 3 (Molecular Devices). EC50 values were determined from dose–responses performed in triplicate, repeated two to four times by subtracting minimum signal from maximum signal during the test period.

### Luciferase assay

U2OS cells were maintained in DMEM containing 4.5 g/L glucose and 10% fetal bovine serum (FBS) supplemented with penicillin-streptomycin. For luciferase assays, U2OS cells were plated in white, clear bottom 96 well plates (Corning Costar 3610) at a density of 25,000 cells per well. Cells were cultured overnight and transfected with 30 ng human FXRalpha2 expression plasmid and 30 ng FXRE driven luciferase reporter plasmid (generously provided by Paul Dawson) using Lipofectamine 3000 reagent according to the manufacturer’s instructions. 30 ng bile acid transporter (NTCP) expression plasmid was transfected in indicated samples. β-galactosidase (β-Gal) was used for normalization. 24 h after transfection, FXR ligands were added in media. Luciferase activity was assayed 24 h after ligand addition. Normalized luciferase values were determined by dividing the luciferase activity by the β-galactosidase activity. Data are presented as fold induction relative to vehicle (DMSO).

### Cell fractionation and mRNA half-life

For nuclear-cytosolic fractionation, HepG2 cells were harvested by trypsinization. Cells were washed and pelleted and the resulting pellet was resuspended in hypo-tonic NP-40 lysis buffer (10 mM Tris-HCl pH 7.4, 10 mM NaCl, 3 mM MgCl2 and 0.5% NP-40) and incubate it on ice for 5 min. Nuclei were pelleted by centrifugation at 1000*g* for 5 min at 4 °C. The supernatant (cytosolic fraction) was saved for RNA extraction. The nuclear pellet was washed with PBS. RNA was extracted from either the nucleus or cytoplasm using Trizol according to the manufacturers’ instructions. Purity (enrichment) was tested using the expression of nuclear restricted ribosomal RNAs by qRT-PCR. For determination of mRNA half-life HepG2 cells were treated with 4 μM actinomycin D dissolved in DMSO for the indicated time intervals. All cells treated with actinomycin D for various periods were harvested at the same time. RNA was extracted using Trizol according to the manufacturer’s instructions.

### RNA extraction & qRT-PCR

RNA was extracted from cells or snap-frozen tissue samples (approximately 50 mg) using Trizol according to the manufacturer’s instructions and as described previously in detail [25]. Liver samples were homogenized before extraction in a MagNA Lyser at 6500 rpm for 2 × 20 s intervals. Concentration and quality (absorbance 260/280 & 230/280 ratios) of RNA was measured on a NanoDrop^™^ 2000 spectrophotometer. Isolated RNA was reverse-transcribed by using the SuperScript II cDNA synthesis enzyme according to the manufacturer’s instructions. Primers used for RT-qPCR were designed using the open-source program Primer3. Primers were designed to span exon-intron boundaries with an amplicon size of less than 150 bp. Relative changes were calculated using a standard curve and 36b4 as the reference genes. Primer sequences are shown in Table 1.

### Liquid Chromatography-Mass Spectrometry (LC-MS) of bile acids

The protocol has been published in detail previously [26]. Briefly, internal standards were added to cells in 12-well plates and cells were lysed in methanol. The debris was pelleted, the supernatant dried and dissolved in running buffer. BAs were separated by HPLC using a C18 reversed phase or pentafluorophenyl column prior to quantification by mass spectrometry.

### Animals, Treatments, CBDL and Tissue Collection

All animals were housed with a 12-h light-dark cycle in humidity and temperature-controlled conditions and permitted *ad libitum* consumption of water and a standard mouse diet (Altromin 1324, Lage, Germany). All the experiments were approved by the European Directive 2010/63/EU and approved by the Austrian Federal Ministry of Education, Science and Research (Vienna, Austria; BMWFW-66.010/0124-V/3b/2018, BMWFW-66.010/0129-WF/V/3b/2016, and the Institutional Animal Care and Use Committees at Emory University and were in conformity with the Austrian/US animal law. The *Ost*α^−/−^, *Asbt*^−/−^, *Ost*α-*Asbt* DKO (double knockout) and *Fxr*^−/−^mice were generated as previously described [20,21]. Experiments were performed in 8–12 weeks-old mice. Mice were fed a chow diet (Altromin 1324, Lage, Germany); for BA supplementation experiments, male mice were fed 0.5% (*w*/w) diet of the indicated BA for the indicated time. CBDL was performed as described previously [25]. Mice were euthanized by decapitation and tissues including liver and intestine were immediately dissected, weighed and frozen in liquid nitrogen for subsequent molecular analysis or preserved for histological analysis.

### Statistical Analyses

Mean values ± SEM are shown unless otherwise indicated. The data were evaluated for statistically significant differences using the Mann–Whitney test, the 2-tailed Student *t-*test, or by analysis of variance (ANOVA, Holmes-Sidak multiple comparisons) (GraphPad Prism 8; Version 8.4.3; Mountain View, CA). Differences were considered statistically significant at *P <* 0.05.

## 4. Results

### IsoBAs are generally weaker FXR agonists than their alpha-epimers

**Fig. 1.**
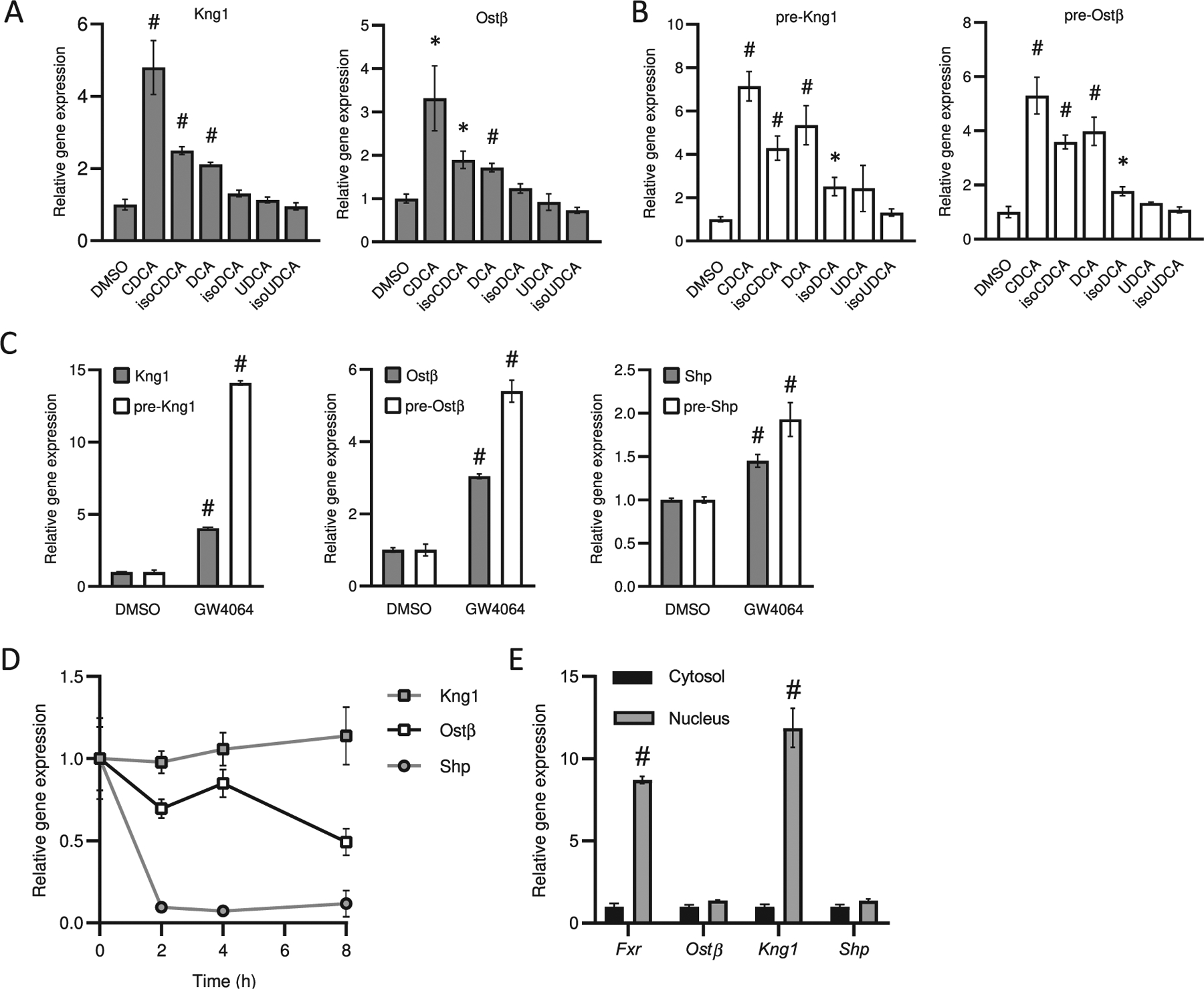
Effect of different isoBAs and corresponding stereoisomers on farnesoid X receptor (FXR) activation. (A) mRNA expression levels and (B) pre-mRNA levels of FXR target genes (*Ost*b, *Kng*1) in HepG2 cells treated for 6 h with 3-alpha hydroxy and corresponding isoBAs. (C) Effect of the synthetic FXR ligand (GW4064) on mRNA and pre-mRNA levels of *Ost*b, *Kng*1, *Shp*. (D) mRNA half-life of FXR target genes and (E) distribution of *Fxr* and its target gene mRNAs (cytosol *vs.* nucleus); *n* = 3–4, of at least two independent experiments. Values represent the mean ± SEM. *p < 0.05, #*p <* 0.01.

We first investigated whether isoBAs activate FXR. Luciferase reporter assays revealed that only isoCDCA showed robust activation of FXR (Suppl. Fig. 1A) In addition, we treated HepG2 cells for 6 h with each of isoCDCA or CDCA, isoDCA or DCA or isoUDCA or UDCA and analyzed FXR target gene expression. Similarly, a significant induction of *Ost*β and *Kng*1 mRNA was observed after 6 h with CDCA *>* isoCDCA, whereas expression of those genes was barely induced by isoDCA and isoUDCA. We therefore also assessed the transcriptional response by quantifying pre-mRNA levels of those target genes. *Ost*β and *Kng*1 pre-mRNAs were robustly induced by CDCA*>* isoCDCA, DCA *>* isoDCA, but not by UDCA, isoUDCA (Fig. 1A, B), CA, isoCA, LCA or isoLCA (data not shown). This more sensitive and dynamic regulation of FXR target gene pre-mRNAs was also observed in HepG2 cells treated with GW4064 (Fig. 1C) as well as in animals treated with the synthetic FXR ligand GSK2324, where Bsep pre-mRNA levels were induced ~20-fold over the mature Bsep mRNA (Suppl. Fig. 2A). The distinct induction of pre-mRNAs as well as the differential regulation in mRNA expression prompted us to investigate the half-life of different FXR target genes and to explore the distribution of those mRNAs between nucleus and cytosol. Recent data suggest that certain spliced mRNAs are retained in the nucleus, including genes that encode transcription factors [27]. The half-life of *Shp* mRNA (~1 h) was much lower compared to *Ost*β (~8 h) or *Kng*1 (*>*8 h) (Fig. 1D). In line with the differences in mRNA half-life, we observed continuously increasing amounts of mature mRNA for *Ostβ* and *Kng1* in HepG2 cells treated with isoBAs over time. Only a short burst induction of all pre-mRNAs was noted. *Shp* mRNA quickly decreased after peaking at 2 h, which is in accordance with its short mRNA half-life (Suppl. Fig. 2B,C). Interestingly *Kng*1 was also highly enriched in the nucleus compared to *Ost*β or *Shp*. Likewise, the mRNA of *Fxr* was also highly retained in the nucleus (Fig. 1E). Our data suggest that it is difficult to measure the dynamic behind FXR-activation using cell-based luciferase assays or determination of mRNA expression by qPCR. IsoBAs are generally weaker FXR agonists compared to their 3α-epimers.

## 5. IsoBAs are rapidly metabolized to 3-keto intermediates and 3α-stereoisomers

IsoBAs, commonly found in the feces of man and animals are not present in bile, because after reabsorption and transport to the liver these molecules undergo rapid re-epimerization to their 3α-stereoisomers [28]. The analysis and quantification of isoBAs *e.g.* isoCDCA and isoDCA can further be challenging, because these isoBAs have similar retention characteristics in reverse phase HPLC [1]. Furthermore, inaccurate use of the trivial name “Isodeoxycholic Acid” for both (5β, 7α, 12α) dihydroxy-cholan-24-oic acid (CAS 566-17-6) and (5β, 3β, 12α) -dihydroxy-cholan-24-oic acid (this study) has led to widespread confusion in the field. Although both are isomers of DCA, this misnomer has led to the use of the incorrect epimer for studies, complicating interpretation of isoDCA measurements in cells, animals and humans [29–32]. We performed liquid chromatography–mass spectrometry (LC-MS) of authentic prospectively synthesized isoBAs on C18 (Suppl. Fig. 3A) and pentafluorophenyl propyl (PFP) columns (Suppl. Fig. 3B) to analyze retention times on both columns. The PFP column allowed us to discriminate isoCDCA from isoDCA.

**Fig. 2.**
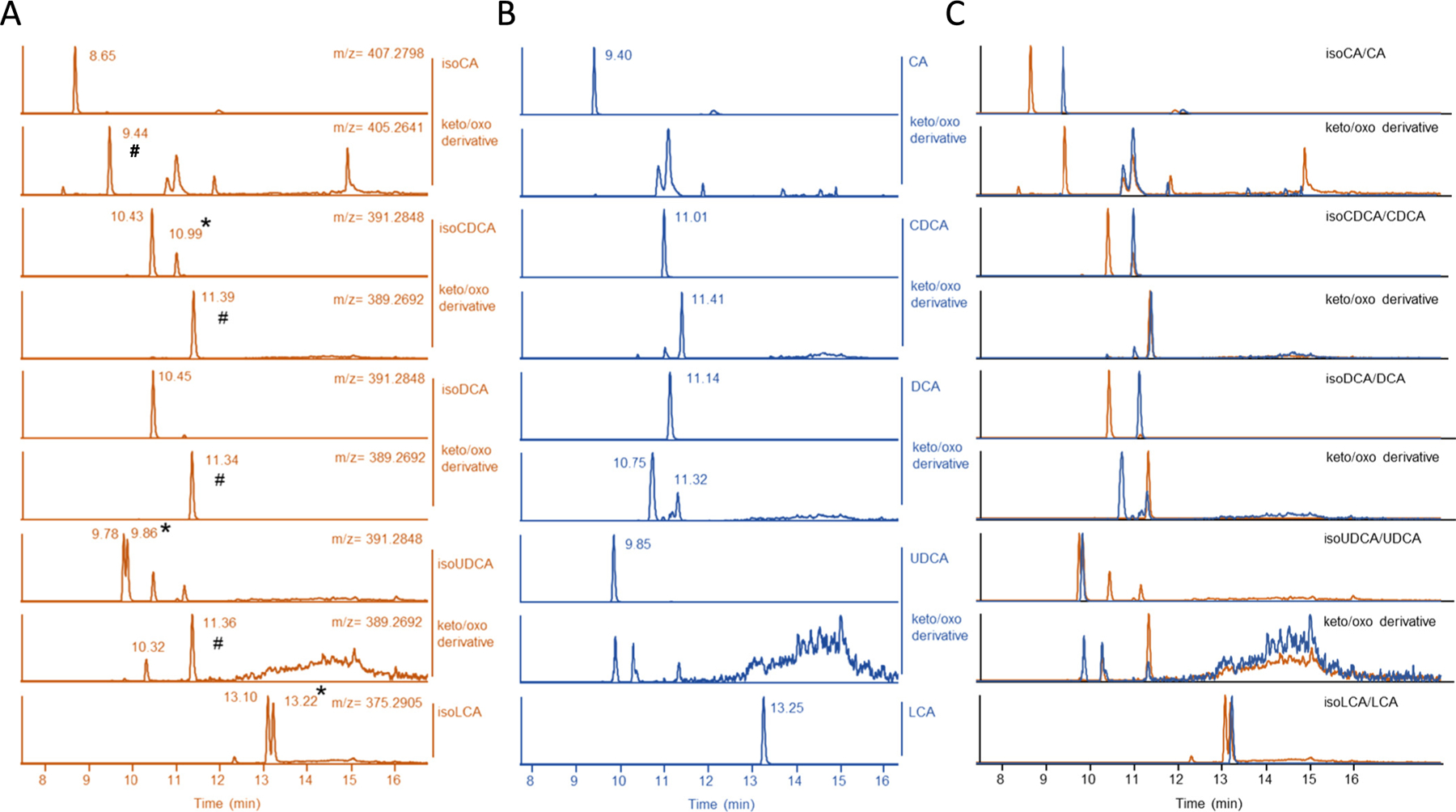
IsoBAs are metabolized into (3-keto/oxo) derivatives and are partially re-epimerized into 3-alpha hydroxy BAs. HepG2 cells were incubated with (A) isoBAs or (B) their 3-alpha-epimers for 4 h. BAs were measured on C-18 column by LC/MS. Upper panel BAs and isoBAs; lower panel keto/oxo derivatives after incubation. Chromatograms of isoBA-standards are shown in Supplementary Fig. 3A. (C) Merged chromatograms of (A) & (B). (#) shows 3-keto/oxo derivatives. (*) conversion of isoBAs into 3-alpha hydroxy BAs. (*m*/*z*) mass divided by charge number.

**Fig. 3.**
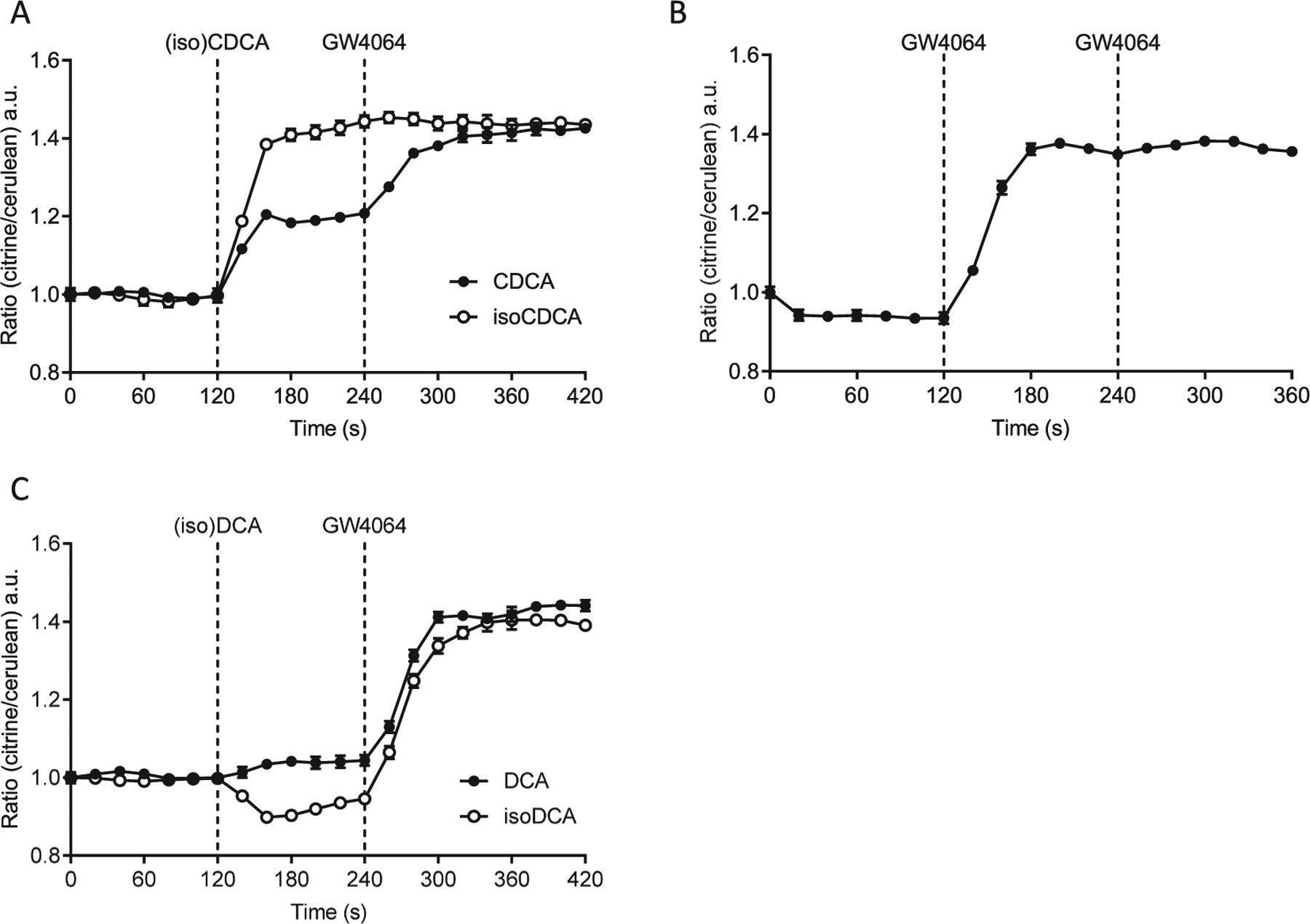
Measurement of FXR activation by isoBAs in real-time using a bile acid sensor (NucleoBAS). (A) Changes in FRET emission ratio upon addition of 100 μM isoCDCA or CDCA at *t* = 120 s and 5 μM GW4064 at *t* = 240 s. (B) Changes in FRET emission ratio upon addition of 5 μM GW4064 alone at t = 120 s and at t = 240 s. (C) Changes in FRET emission ratio upon addition of 100 μM isoDCA or DCA at t = 120 s and 5 μM GW4064 at t = 240 s. (*n* = 6 cells per experiment). At least three independent experiments were performed; Error bars represent SEM.

Having established suitable analytical methods, we focused on the metabolism of isoBAs and their 3-alpha-epimers *in vitro*, using conditions similar to our cell-based FXR assays. Surprisingly, liquid chromatography analysis of isoBAs extracted from HepG2 cells treated for only 4 h revealed that a significant amount of isoBAs undergoes metabolism to the corresponding 3-keto intermediates (# in Fig. 2A) and 3α-stereoisomers (* in Fig. 2A). Conversion of isoCDCA into CDCA or isoUDCA into UDCA was observed (Fig. 2A–C). Similar results were obtained for isoCA and isoLCA (Fig. 2A, C). Although we found keto/oxo derivatives of isoCA and isoDCA, we did not observe re-epimerization of those isoBAs into their 3α-stereoisomers. This may indicate that the presence of the 12-hydroxy group prevents further metabolism into 3α-BAs. It was also observed that a very small amount of 3α-OH BAs were converted to 3-keto intermediates (Fig. 2B, C). These results indicate that hydroxysteroid dehydrogenases and 3-oxo-reductases quickly metabolize iso-BAs in HepG2 cells in culture, precluding use of this system to study the direct actions of isoBAs.

## 6. IsoBAs show marked modulatory effects on nuclear FXR

The rapid metabolism of isoBAs and their 3α-OH epimers makes it demanding to study and interpret their impact on FXR activation. Previously, we developed a genetically encoded “Bile Acid Sensor” (NucleoBAS) containing cerulean and citrine as the donor and acceptor that form a Förster Resonance Energy Transfer (FRET) pair. The FRET Bile Acid Sensor further contains the ligand binding domain (LBD) of FXR and a peptide derived from nuclear receptor coactivator 2 (NCoA2), which binds FXR-LBD in the presence of BAs. This sensor generally works like a TR-FRET FXR coactivator assay, but can directly monitor BA binding to FXR within seconds in single live cells [22,23].

Using this system we examined, whether isoBAs act as FXR agonists and lead to coactivator recruitment on a short timescale. Firstly, several isoBAs and their 3α-epimers were analyzed for their ability to induce FRET in NucleoBAS expressing cells. NucleoBAS expressing cells incubated with isoCDCA exhibited increased FRET, even higher as compared to CDCA (Fig. 3A). Subsequent addition of the synthetic FXR agonist GW4064 did not further increase the citrine to cerulean emission ratio in cells pretreated with isoCDCA, suggesting that the maximum response of the NucleoBAS was already reached at this dose of isoCDCA. In contrast, cells pretreated with a similar dose of CDCA still showed increased FRET after addition of GW4064, indicating submaximal stimulation by CDCA alone. Treatment with GW4064 alone led to a similar increase in FRET compared to BA pretreated cells. FRET remained steady upon administration of a second addition of GW4064, indicating that the maximum response of the sensor has been reached (Fig. 3B). DCA treatment led to minimal increase of FRET (Fig. 3C). However, the addition of isoDCA decreased the emission ratio, indicating that binding of isoDCA to the sensor induces conformational changes that differ from canonical BA-signaling.

## 7. Secondary isoBAs reduce FRET emission ratios in the presence of endogenous BAs

**Fig. 4.**
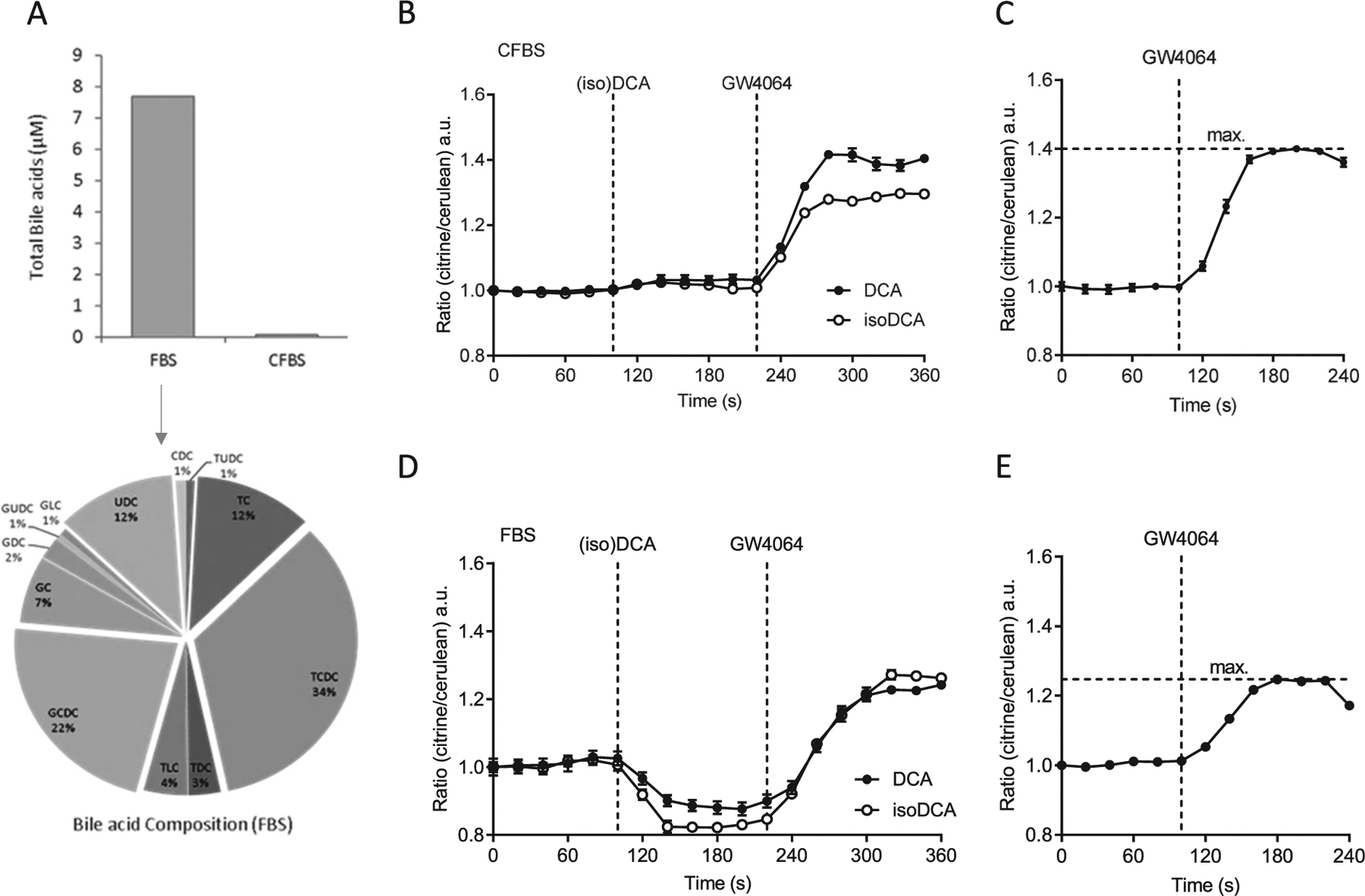
FRET emission ratios in NucleoBAS expressing cells cultured in DMEM containing complete FBS (+FBS) or charcoal stripped FBS (+CFBS). (A) Quantification of total BAs from FBS and CFBS and composition of individual BAs shown as percentage; UDC = Ursodeoxycholic, C = Cholic, CDC = Chenodeoxycholic, DC = deoxycholic, LC = Lithocholic; G (glycine) and T (taurine) conjugated bile acids. (B-E) Changes in FRET emission ratio in NucleoBAS cells expressing NTCP-mKate2 cultured (B) in DMEM containing **CFBS** upon addition of 100 μM isoDCA or DCA at *t* = 100 s and 5 μM GW4064 at *t* = 220 s. (C) Changes in FRET emission ratio of cells cultured in DMEM containing **CFBS** upon addition of 5 μM GW4064 alone at *t* = 100. (D) Changes in FRET emission ratio of cells cultured in DMEM containing **FBS** upon addition of 100 μM isoDCA or DCA at t = 100 s and 5 μM GW4064 at t = 220 s. (E) Changes in FRET emission ratio of cells cultured in DMEM containing **FBS** upon addition of 5 μM GW4064 alone at t = 100. (n = 6 cells per experiment). Error bars represent the SEM.

Induced FRET emission rations lower than baseline had not been observed previously [22,23]. We hypothesized that isoDCA displaces BAs present in fetal bovine serum (FBS), which are also responsible for the apparently activated state at *t* = 0. FBS contains numerous BAs (Fig. 4A) with glycine and taurine conjugated chenodeoxcholic and cholic acid (G/T-CDC, -CA) as well as unconjugated ursodeoxycholic acid (UDC) being most abundant (Fig. 4A lower panel). These are essentially absent in charcoal stripped FBS (CFBS). Therefore, NucleoBAS expressing U2OS cells were cultured in medium containing CFBS to minimize potential background effects of BAs found in FBS on the ligand-binding domain of the sensor. Under these conditions, isoDCA and DCA did not depress FRET below baseline (Fig. 4B). Next, NTCP-expressing NucleoBAS cellswere cultured in medium supplemented with full serumto allow entry of both conjugated and unconjugated BAs found in FBS, in order to mimic aspects of an *in vivo*-like environment. IsoDCA and to lesser extend DCA again led to a reduction of FRET below baseline (Fig. 4D). UDCA and isoUDCA (Suppl. Fig. 4A, B), secondary (iso)BAs that are found in mice and humans, showed similar effects. Notably, addition of GW4064 by itself produced a higher maximal response in CFBS (Fig. 4C) than in FBS (Fig. 4E). It is interesting to note that in all experiments cells pre-incubated with either isoDCA or DCA, even if BAs from FBS were still present in the cell, more rapidly reached the maximal change in emission ratio after addition of GW4064 and a maximal response similar to cells treated with GW4064 alone (Fig. 4B, D).

Next, we investigated whether secondary isoBAs can decrease emission ratios induced by a single BA. We used glycochenodeoxycholic acid (GCDCA), a BA known to activate FXR. NTCP-NucleoBAS expressing cells treated with GCDCA in CFBS markedly increased FRET. At *t* = 240 s, isoDCA as well as isoUDCA, reduced the FRET signal (Suppl. Fig. 4C) suggesting that both secondary BAs can exert this effect in the presence of a single FXR agonist.

In summary, our data indicate that naturally occurring BAs present in FBS might cause partial saturated and activated FXR in our nuclear Bile Acid Sensor set-up. Secondary BAs such as (iso)DCA and (iso)UDCA can reverse ligand-induced conformational changes of NucleoBAS (= co-activator peptide recruitment) but those conformational changes towards suppression of receptor activity do not interfere with a second stimulatory response of FXR.

## 8. Secondary isoBAs act cooperatively with FXR ligands *in vivo*

In our *in vitro* experiments, we compared the effects of 3α- and 3β-epimers of different BAs on FXR activity and target gene expression. We further aimed to evaluate their impact *in vivo* using C57BL6/J mice. In our FXR-Sensor experiments, the presence of BAs from FBS interfered with the activation of FXR by GW4064 (Fig. 4E). In addition, isoBAs are immediately metabolized to 3-keto-intermediates. In human cultured enterocytes, that do not metabolize isoBAs, the presence or absence of isoBAs in combination with steroidal or non-steroidal FXR agonists showed no significant additive effect on the expression of *Fgf19* and *Ibabp*, *in vitro* (Suppl. Fig. 1C). We therefore administered isoBAs (isoUDCA and isoDCA) and their stereoisomers (UDCA and DCA) followed by GW4064, *in vivo*.

**Fig. 5.**
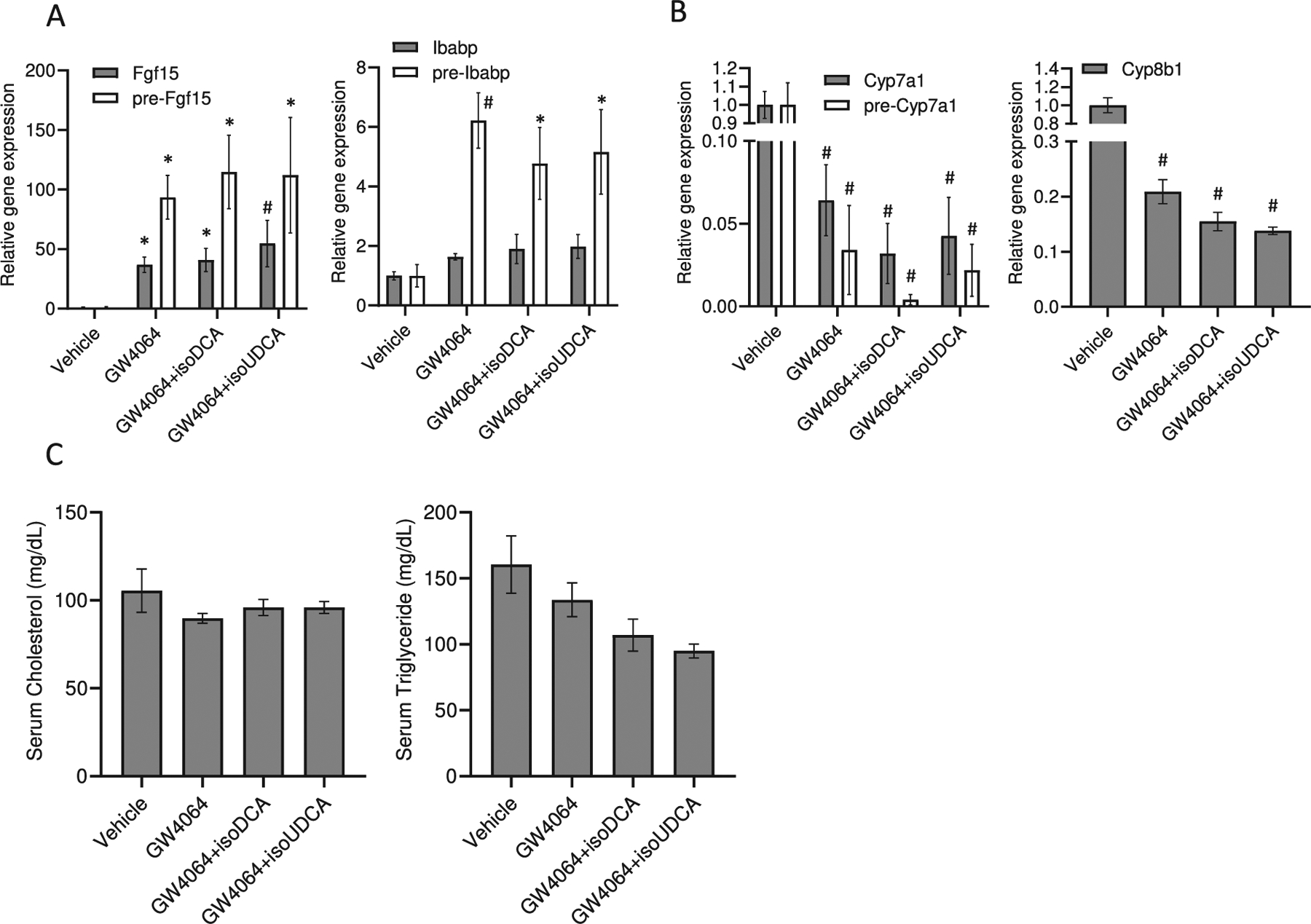
Regulation of FXR target genes *in vivo* by single dose administration of secondary isoBAs and GW4064. (A) Regulation of *Fgf*15 and *Ibabp* pre-mRNA and mRNA by GW4046, isoDCA+GW4064 and isoUDCA+GW4064. (B) Repression of bile acids synthesis genes (*Cyp*7a1 and *Cyp*8b1) in livers of mice treated with isoBAs and GW4064 as well as (C) serum cholesterol and triglyceride levels. Administration of isoBAs (isoUDCA and isoDCA) followed by GW4064 with 1 h time-delay for a total of 6 h. Mice are gavaged with olive oil (vehicle), GW4064 (30 mg/kg BW), and isoBAs (60 mg/kg BW). Genes were analyzed 6 h after gavage; n = 3–4, values represent the mean ± SEM. *p *<* 0.05; #p *<* 0.01.

As illustrated in (Fig. 5A), co-treatment caused a trend to additively induced *Fgf*15 pre-mRNA and mRNA expression, which was of course already remarkably enhanced by GW4064. Ileal bile acid binding protein (*Ibabp*) pre-mRNA was not affected by pre-treatment with isoBAs (Fig. 5A) similar to *Ost*β, which was also already strongly induced by GW4064 (data not shown). There was no antagonistic effect of both isoBAs noted.

**Fig. 6.**
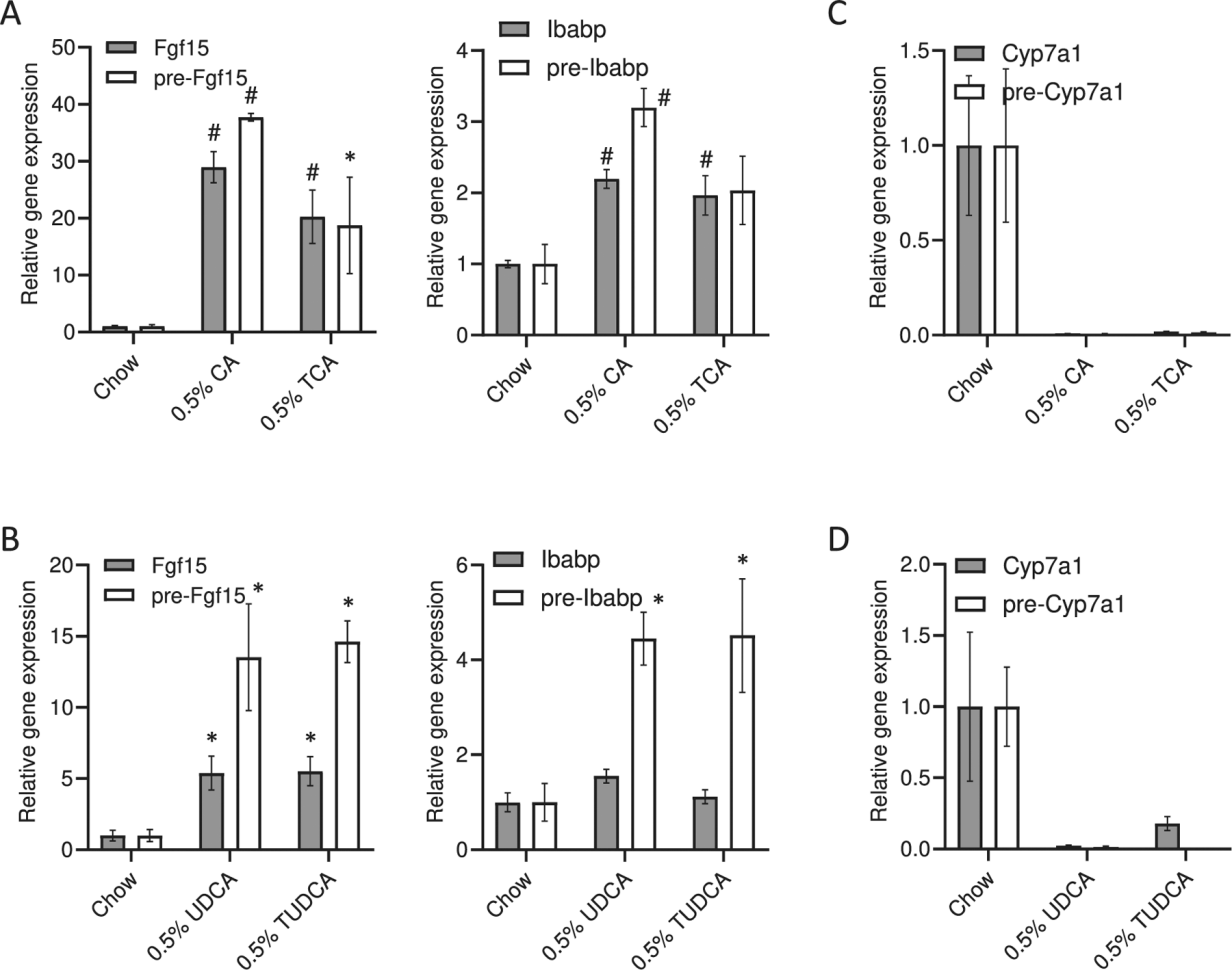
Regulation of FXR target genes *in vivo* after treatment with unconjugated and conjugated bile acids. C57BL/6 J mice were treated with cholic (CA) and taurocholic (TCA), ursodeoxycholic (UDCA) and tauroursodeoxycholic (TUDCA) acid 0.5% (*w*/w) chow diet. (A-B) Regulation of intestinal *Fgf*15 and *Ibabp* pre-mRNA and mRNA by CA, TCA, UDCA, and TUDCA. (C–D) Repression of hepatic bile acids synthesis genes (*Cyp*7a1 and *Cyp*8b1) by CA, TCA, UDCA, and TUDCA. *n* = 4–5, values represent the mean ± SEM. *p *<* 0.05; #p *<* 0.01.

Even though, there was only a slight cooperative regulation on the already very strong effect of GW4064 in the ileum, in the liver, isoBAs and GW4064 showed a robust additive effect on the repression of *Cyp*7a1 mRNA and pre-mRNA and *Cyp*8b1 mRNA (Fig. 5B). Serum triglyceride but not cholesterol levels in co-treated animals tended to be lower (Fig. 5C). Our data indicate that a single dose of isoBAs within 6 h results in a modest synergistic effect with the synthetic FXR ligand *in vivo*. Since isoBAs are quickly metabolized to 3-oxo/3α-hydroxy BAs in the liver and re-secreted again as conjugated BAs into the intestine we were bound to restrict our measurements to a narrow time window of max. 6 h. Furthermore, we conducted an experiment to directly compare UDCA *vs.* isoUDCA and DCA *vs.* isoDCA in the context of selective FXR activation with GW4064 (Suppl. Fig. 5A,B). There were no major differences between isoUDCA/UDCA in the intestine (maybe a slightly better activation by isoUDCA). In the liver, the repression of Cyp7a1 was even better with isoUDCA compared to UDCA (Suppl. Fig. 5B). Due to the laborious synthesis steps required and costs to acquire sufficient amounts of isoBAs for an extended experiment *in vivo*, we fed mice with either free or conjugated UDCA or *CA.* In our previous FXR sensor experiments, the effects of UDCA were comparable to those seen with isoUDCA. Cholic acid (CA), which is metabolized to DCA *in vivo* in the intestine, was used for comparison, acting as a direct FXR ligand. DCA was not administered directly since it has been reported to be hepatotoxic [33]. CA- and UDCA-enriched diets both increased *Fgf*15 and *Ibabp* expression in the intestine (Fig. 6A, B) and repressed *Cyp*7a1 levels in the liver (Fig. 6C, D). Notably, UDCA as well as TUDCA more strongly enhanced the expression of pre-mRNA over mRNA as compared to CA and TCA, indicating a short rather than constant burst of FXR target gene expression.

## 9. UDCA exerts FXR agonistic activity in the presence of endogenous bile acids

**Fig. 7.**
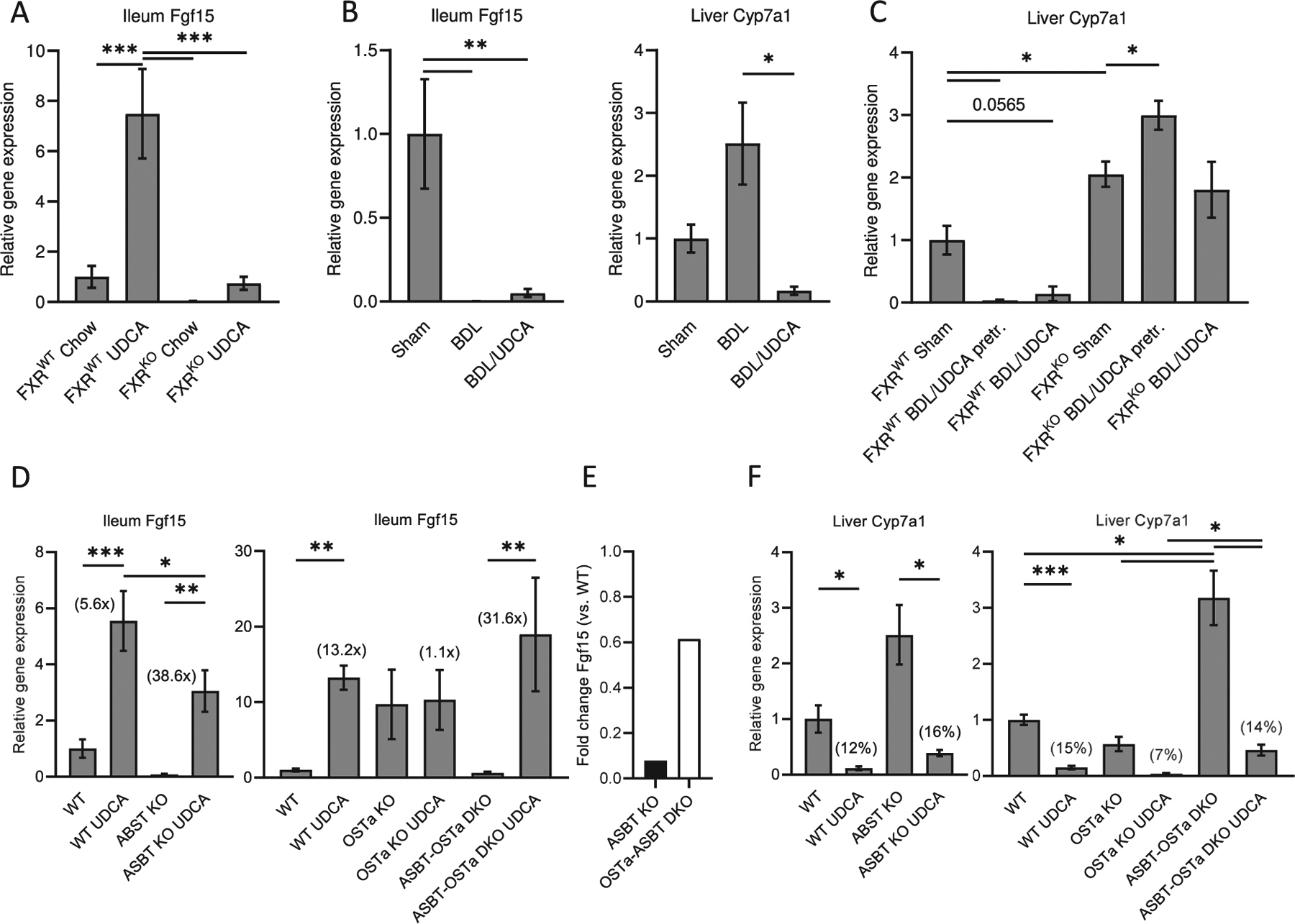
Regulation of intestinal *Fgf*15 and hepatic *Cyp*7a1 by ursodeoxycholic (UDCA) related to an intact enterohepatic circulation. (A) C57BL/6 J mice (FXR WT) and *Fxr* knockout (FXR KO) animals were fed either chow or ursodeoxycholic (UDCA) 0.5% w/w diet for 7 days. (B) C57BL/6 J mice were either sham operated (Sham) or subjected to common bile duct ligation (BDL) with or without UDCA 0.5% (w/w) enriched diet for 7 days. (C) Experiments performed like in (B) using FXR WT and FXR KO mice pretreated with UDCA for 3 days before BDL or co-administered UDCA with BDL for 7 days. (D–F) Administration of 0.5% (w/w) UDCA enriched diet for 7 days in WT and knockout (KO) mice, including *Asbt* KO, *Ostα* KO and *Asbt*-*Ostα* DKO. n = 6–7, values represent the mean ± SEM. *p *<* 0.05, **p *<* 0.01, ***p < 0.001.

UDCA is able to increase serum FGF19 and reduce levels of 7α-hydroxy-4-cholesten-3-one (named C4), an intermediate product and marker of BA synthesis [34]. However, although UDCA binds to FXR, it shows no to weak direct agonistic activity [35], indicating that UDCA is a FXR activator, but in general is not as effective as CA or DCA [36]. We next wanted to gain insights into the molecular basis of FXR signaling in cholestatic liver diseases, especially the role of FXR in mediating the effect of UDCA on genes related to BA-synthesis. We administered UDCA to *Fxr* WT and KO mice and to animals with common bile duct ligation (CBDL), which results in a lack of endogenous BAs in the intestine. Administration of UDCA strongly increased *Fgf*15 in WT mice, but not in *Fxr* KO mice (Fig. 7A). Notably, UDCA was unable to increase *Fgf*15 in CBDL mice (Fig. 7B), indicating that endogenous BAs have to be present in the intestine to observe FXR activation. In the liver, UDCA still inhibited *Cyp*7a1 mRNA expression (Fig. 7B). These data argue for an FXR-dependent effect of UDCA in cholestatic animals, with high BA levels present in the liver.

Of note, while CBDL disrupts enterohepatic circulation and thereby inhibits BA and FXR signaling in the intestine, endogenous BAs and FXR are still present in the livers of those animals. Treatment with UDCA in CBDL-WT but not CBDL-*Fxr* KO mice did repress *Cyp*7a1 mRNA levels (Fig. 7C). Our data indicate that decrease of *Cyp7a1* mRNA in CBDL animals treated with UDCA depends on hepatic FXR and could be related to a rapid degradation of *Cyp*7a1 mRNA by posttranscriptional mechanisms [37].

Pruritus represents a multifaceted symptom in cholestatic liver diseases. Among many proposed mechanisms, it seems to be causally linked to the activation of the G protein-coupled receptors (GPCRs). Besides FXR, secondary BAs strongly activate two GPCRs, TGR5 and MRGPRX4, both associated with itch in cholestatic liver disease. isoBAs are generally weaker agonists for TGR5 compared to their 3-alpha-epimers [38]. Based on this background we aimed to investigate the role of isoUDCA and isoDCA on MRGPRX4 signaling, which is sufficient to trigger itch sensation in humans. Our data demonstrate that both isoBAs were weaker agonists than their corresponding 3α-epimers: isoUDCA has a 7-fold higher EC_50_ on MRGPRX4 compared to UDCA, whereas isoDCA has a 3-fold higher EC_50_ compared to DCA (Suppl. Fig. 6 A, B) suggesting isoBAs are less potent agonists of MRGPRX4 compared to 3α-BAs. These data could argue for a potential advantage of isoBAs over 3α-OH BAs in the treatment cholestatic liver diseases associated with itch.

To further investigate how UDCA in cooperation with endogenous BAs affects FXR activity, we used mice deficient for the intestinal BA-transporter (IBAT, also known as Apical Sodium Bile Salt Transporter, ASBT). Intestinal BA malabsorption in *Asbt*-KO mice is associated with a significant increase in hepatic *Cyp*7a1 mRNA, as well as almost undetectable levels of ileal *Fgf*15 mRNA levels (Fig. 7D, E). Administration of UDCA in *Asbt*-KO mice rescued the low levels of intestinal *Fgf*15 mRNA but was not able to reach WT levels (Fig. 7D). Contrary to *Asbt* KO, *Ost*α knockout (*Ost*α-KO) mice served as a model of continuous intestinal FXR activation by trapping BAs in the enterocytes [21]. In this mouse model, UDCA was unable to further stimulate supersaturated FXR and to induce *Fgf*15 mRNA levels (Fig. 7D). The inability of endogenous BAs to escape from the enterocyte by OSTα deficiency combined with the reduced uptake of conjugated BAs by ASBT deficiency (*Ostα*-*Asbt* DKO mice) seems to reconstitute FXR activity close to baseline in the ileum of this unique DKO mouse model (Fig. 7E). Notably, in DKO mice enterocytes UDCA robustly stimulated *Fgf*15, but this time reaching levels similar to treatment in WT animals (Fig. 7D). In all mouse models studied UDCA repressed hepatic *Cyp*7a1 levels to a similar extend as in WT mice (Fig. 7F). These findings clarify an important role of BA-transport proteins on balanced FXR-signaling within the enterohepatic circulation and suggest that UDCA regulates BA metabolism *via* indirect agonistic behavior on FXR in liver and intestine.

## 10. Discussion

In summary, our data demonstrate that different isoBAs as well as their 3α-epimers have unique properties as FXR ligands, ranging from agonist, to partial agonist or ago-allosteric modulators. The relationship between structural aspects of BAs with regard to their C3-OH orientation and FXR activation has been previously investigated; 3β-epimers of DCA and LCA activated FXR, although to a much lower extend than their 3α-epimers [11,39]. We also observed that the 3β-orientation of BAs significantly blunted FXR activity in gene expression and luciferase assays. Notably, our analysis of cellular extracts demonstrated that after 4 h a significant amount of isoBAs was already metabolized to the corresponding 3-keto intermediates and further into 3α-epimers (Fig. 2A). Extended incubation times (~20 h) will therefore led to a mixture of BA intermediates and 3α-epimers and do not allow for direct effects of isoBAs to be studied. Remarkably, discrepancies regarding FXR activity between results obtained from cell-based reporter assays and *in vitro* co-activator recruitment assays are often observed [39,40]. One explanation could be the difference in incubation time for these assays: ~24 h for cell based *vs.* ~1–2 h for *in vitro* coactivator association assay.

Besides coactivator recruitment and chromatin modifications, target gene expression levels depend on processing of pre-mRNA as well as mRNA half-life and localization for a given gene of interest. We show that isoCDCA and CDCA were the most potent FXR agonists in cell-based luciferase assays and effectively regulated expression of *Kng*1 and *Ost*β, both well-known FXR target genes, in HepG2 cells. The level of target gene induction seemed to positively correlate with half-life and nuclear localization of its mRNA, as stimulation by GW4064 similarly to stimulation by BAs increased *Kng*1 *> Ost*β *> Shp* mRNA. Recently it has been demonstrated that temporal resolution of these rates can be inferred from simultaneous measurements of precursor mRNA (pre-mRNA) and mature mRNA profiles [41]. Single-nuclei RNA-seq is now emerging as a tool to characterize cell specific effects under various physiological and pathological conditions. Somewhat similar to our approach, this method covers preferentially pre-mRNAs and mRNAs with nuclear enrichment, and may lead to the discovery of new FXR target genes. Therefore, the analysis of pre-mRNA, especially in short incubation experiments is of particular importance to observe the early activation of FXR.

However, our initial methods to explore FXR activation only provide a signal that is (i) averaged over many cells, which are (ii) treated for a long period, and (iii) allow only endpoint measurement of activation of the transcriptional machinery. The use of our genetically encoded fluorescent sensor (NucleoBAS) allows the imaging of BAs on FXR activation within seconds at a single cell level, and in parallel, monitoring of *e.g.* BA-uptake from complex media. Using this tool we were able to investigate the effects of isoBAs on FXR-LBD conformational changes and co-activator recruitment in real-time. NucleoBAS expressing cells incubated with isoCDCA exhibited increased FRET efficiency even higher than CDCA. Astonishing however was the observation that addition of secondary isoBAs, isoUDCA and isoDCA even decreased the emission ratio, indicating that binding of these BAs to the sensor induces conformational changes opposite to canonical BA-signaling. It should also be mentioned that co-expression of a bile acid uptake transporter protein is required for efficient uptake of relatively hydrophilic BAs; this results *e.g.* in an activation of FXR by otherwise inactive cholic acid and strongly potentiates the effect of DCA on FXR activity [38]. Similarly, the conformational changes by isoBAs only occurred if a BA-transporter and endogenous BAs found in FBS were present; most importantly, this conformational change did not interfere with a second stimulation of FXR.

## 11. What could explain the modulatory effects of secondary (iso)BAs?

A ligand may interact with an allosteric binding pocket, which would be distinct from the classical FXR-LBD binding site. These ligands have the potential to both function as (partial) agonists on their own and to modulate binding as well as signaling of an orthosteric ligand. Such molecules would be called “ago-allosteric modulators”. In line with this reasoning, the identification of a potential second binding site in the FXR-LBD might explain specific differences observed with various selective FXR modulators or different bile acids [42–44]. An example for ago-allosteric modulation refers to UDCA’s binding to one of IBABP’s two BA-binding sites. By doing so, it significantly increases the affinity of the protein for other major FXR-agonistic BAs [45]. However, this effect might be more relevant *in vivo*, as experiments performed with our FXR sensor *in vitro* do not rely on IBABP expression [22].

In addition, when a receptor’s LBD is occupied by ligand 1, the rate of binding and activation by a new ligand (ligand 2) depends on the relative affinity of the two agonists for the receptor and/or the dissociation rate for the first. In theory, if the dissociation rate for ligand 1 is low (high affinity), the activation induced by a new agonist (ligand 2) can be expected to be weak. This model may explain the saturated FXR-signaling in *Ost*α knockout mice and the failure of UDCA to stimulate FXR.

An important question that could only be partially addressed in this study is the *in vivo* long-term effect of secondary isoBAs.

Administration of a single dose of isoBAs alone did not affect the expression of FXR target genes in liver or intestine within 6 h (data not shown). Nevertheless, sequential administration of secondary isoBAs followed by GW4064 caused a trend induce *Fgf*15 expression in the intestine, and resulted in a robust additive effect on the repression of *Cyp*7a1 and *Cyp*8b1 expression. The limited treatment time after the bolus may explain the mild additive effects observed on the already robust induction of FXR signaling by co-administration with GW4064. Importantly, isoBAs were found to have a much weaker affinity for human ASBT compared to native BAs, with the exception of UDCA and isoUDCA, showing identical binding affinities and would therefore not interfere with endogenous BA-uptake [46].

We decided to feed (T-)UDCA and (T-)CA for two main reasons: i) isoBAs are known to be quickly metabolized to 3-oxo/3α-hydroxy BAs in the liver, and re-secreted, again as conjugated BAs, into the intestine. ii) The dose of TCA or TUDCA used in previous mice experiments ranged from 50 mg–500 mg/kg/d over several days [17,47,48], which we could not achieve with isoBAs due to limited amounts of isoDCA and isoUDCA. Our *in vivo* experiments clearly demonstrate that UDCA is associated with FXR agonistic activity, but only in the presence of endogenous BAs. Neither in *Fxr* KO mice, nor in bile duct-ligated animals, UDCA was able to induce *Fgf*15 (Fig. 7A, B). Most intriguing was the effect of UDCA in an animal model of BA-malabsorption (*Asbt* KO mice). Administration of UDCA in *Asbt*-KO mice rescued the low levels of intestinal *Fgf*15 mRNA, but was unable to reach WT expression levels (Fig. 7D). This indicates that in contrast to CBDL small amounts of unconjugated BAs are still sufficient to cooperate with UDCA in *Asbt* KO enterocytes. Moreover, this effect may also be related to the observation that in *Asbt*-KO mice the BA pool is still enriched in cholic acid (FXR agonistic) over muricholic acids (FXR antagonistic), though the total pool size comprising only ~20% of WT animals. Contrary to *Asbt* KO, UDCA was unable to further stimulate intestinal FXR activity in *Ost*α KO mice, because of high levels of endogenous BAs retained in the enterocytes. The inability of endogenous BAs to escape from the enterocyte by *Osta* deficiency, resulting in supersaturated FXR, was reversed by ASBT deficiency (Fig. 7E). Most importantly, balanced concentrations of BAs in DKO enterocytes (a combination of reduced uptake of BAs by ASBT deficiency and the inability of endogenous BAs to escape from the enterocyte by OSTa deficiency) allowed UDCA to stimulate FXR and induce *Fgf15*, but this time to a similar extend as in WT animals (Fig. 7D). Taken together endogenous BAs - not too high or too low - are necessary to facilitate proper activation of FXR by UDCA.

## 12. What could be an advantage of using isoBAs over 3α-epimers?

The direct administration of isoBAs would i) bypass the prolonged biosynthetic steps by bacteria in the intestine, with a selective and higher enrichment of isoBAs, ii) might lead to lower cytotoxicity for host and microbiota. IsoBAs have lower detergent activity and toxicity on bacteria [10], and as our data demonstrate are less toxic to the human epithelial cell line Caco-2 (Suppl. Fig. 1B). IsoBAs also show less cytotoxic effects on human hepatoblastoma or rat intestinal epithelial cell lines [19,49]. One single study has directly compared isoUDCA and UDCA [50]. In rats with an intact enterohepatic circulation, a reduction of total serum BAs was only observed with isoUDCA but not UDCA. This reduction was due to a pronounced decrease of CA, beta-MCA, and DCA as compared to UDCA. Importantly, all these effects disappeared after bile duct ligation. To what extend FXR-signaling was involved in mediating the effect of isoUDCA was not investigated at that time [50].

UDCA has been used for many years to treat liver diseases, such as primary biliary cholangitis (PBC), but also exerts extra-hepatic therapeutic effects like in patients with inflammatory bowel disease. Pilot studies showed that isoUDCA was similarly well tolerated without any side effects [49,51]. Furthermore, isoUDCA was identified in patients with cholestatic liver disease during UDCA therapy. Our data demonstrate that isoUDCA has a 7-fold lower potency compared to UDCA on MRGPRX4, similarly to TGR5, which is also less sensitive to isoBAs [38]. This finding could be further considered for potential equal efficacy but less adverse effects of a treatment with isoUDCA in pruritus-related cholestatic liver disease linked to the activation of TGR5 and MRGPRX4, GPCRs found on itch sensory neurons [24,52].

## 13. Conclusions

Our data suggest that secondary (iso)BAs interact with FXR in a unique fashion that leads to cooperative effects in the presence of endogenous BAs or synthetic FXR ligands to stimulate gene transcription. To what extend patients with PBC are profiting from an UDCA-pretreatment on top of *e.g.* the synthetic FXR ligand obeticholic acid (OCA) remains elusive, but synergistic treatment mechanisms may explain the significant additional efficacy seen when OCA or a low dose of a nonsteroidal FXR agonist was combined with UDCA [53–55].

## Supplementary Material

Supplementary Material

## Figures and Tables

**Table 1:** Primers used in this work.

Name	fwd (5’- > 3’)	rev (5’- *>* 3’)
mFgf15 premRNA	CAT GCA CAC AGC CTC TTG TC	TCC ATT TCC TCC CTG AAG GT
mCyp7a1 premRNA	TTT GCA TCA TGG CTT CAG AG	TCT TCC AGA CAC TCC CCA AC
mIbabp premRNA	GTT CAT TAG GCC GTG ACT AGA G	CTC CGA AGT CTG GTG ATA GTT G
mBsep premRNA	CCTTTGATACCTGGATCCTACTTG	CCTGCGTAGATGCCAGAAA
mFgf15	GAG GAC CAA AAC GAA CGA AAT T	ACG TCC TTG ATG GCA ATC G
mCyp7a1	CAG GGA GAT GCT CTG TGT TCA	AGG CAT ACA TCC CTT CCG TGA
mIbabp	CCC CAA CTA TCA CCA GAC TTC G	ACA TCC CCG ATG GTG GAG AT
mBsep	GAG TGG TGG ACA GAA GCA AA	TGA GGT AGC CAT GTC CAG AA
mCyp8b1	TTA AGG CTG GCT TCC TGA GC	TCG ACG GAA CTT CCT GAA CAG
hOstb premRNA	TGG AGC TAG AAC ACA AGA TCA C	CTA TCT GCC TAC CTA CCC ATC TA
hShp premRNA	AGC CCT CTT CTC CCT CTC	CTT CAC ACA GCA CCC AGT
hKng1 premRNA	CCA GTT TGT ATT GTA CCG CAT AAC	GCC TGG ACT CCT AGT GAT AGT A
hOstb	CAG GAG CTG CTG GAA GAG AT	GAC CAT GCT TAT AAT GAC CAC CA
hShp	GCT TCA ATG CTG TCT GGA GTC	CTT GGA GGC CTG GCA CAT C
hKng1	TTC TGA GAC ACG GCA TTC AG	AAG TTC AAT CCA GCC ACC AC
hFxr	AGG GGT GTA AAG GTT TCT TCA GGA	ACA CTT TCT TCG CAT GTA CAT ATC CAT
36b4	GCT TCA TTG TGG GAG CAG ACA	CAT GGT GTT CTT GCC CAT CAG

## Abbreviations:

ASBT: apical sodium-dependent bile acid transporter
BA: bile acid
CA: cholic acid
CBDL: common bile duct ligation
CDCA: chenodeoxycholic acid
FXR: farnesoid X receptor
HCC: hepatocellular carcinoma
IBD: inflammatory bowel disease
isoBA: iso-bile acid
LCA: lithocholic acid
MCA: muricholic acid
MRGPRX4: Mas-related G-protein coupled receptor member X4
PBC: primary biliary cholangitis
OST α/β: organic solute transporter alpha/beta
UDCA: ursodeoxycholic acid
