# Supplementary material for: Secondary (iso)BAs cooperate with endogenous ligands to activate FXR under physiological and pathological conditions
Source: Biochim Biophys Acta Mol Basis Dis. Author manuscript; Available in PMC 2021 Aug 1. (PMC8177068; doi:10.1016/j.bbadis.2021.166153)
Supplement: Supplementary Material [file NIHMS1706919-supplement-Supplementary_Material.pdf]

A

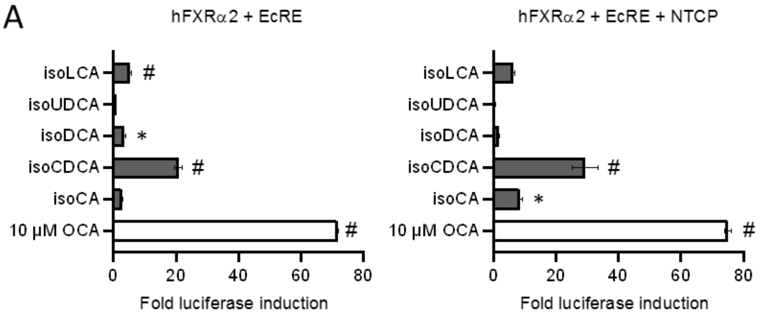

B

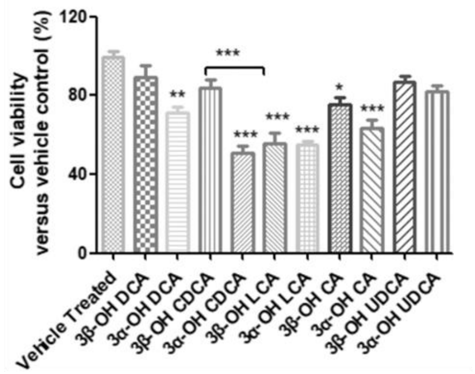

C

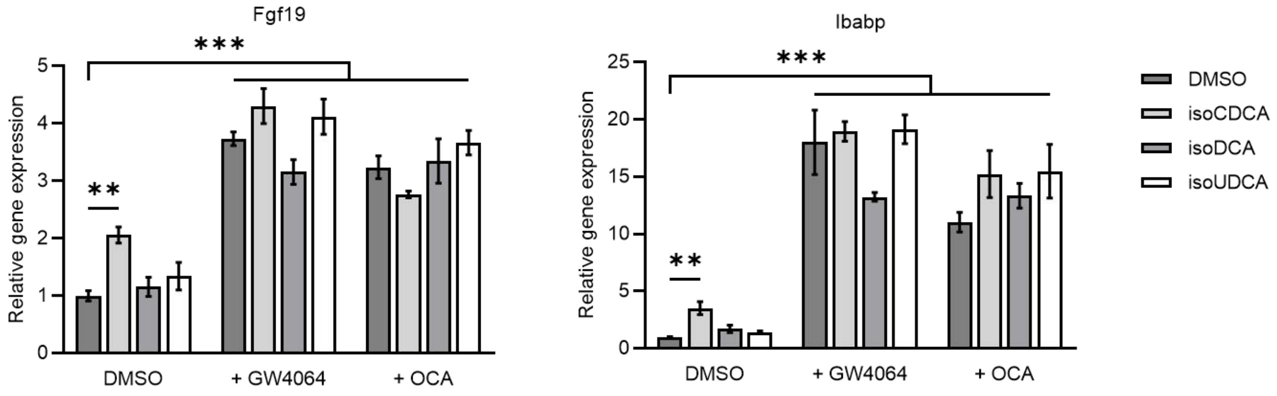

Supplementary Figure 1. (A) Cell-based hFXRa2 luciferase assay co-transfected with or without NTCP; cells were exposed to vehicle (DMSO) or 100µM iso-bile acids (isoBAs). After 24 h of treatment, cells were lysed for determination of luciferase and β-galactosidase activities as described under “Materials and Methods”. (B) Toxicity of isoBAs and corresponding stereoisomers. Caco-2 cells were treated with 100 µM bile acids for 24 h. The cytotoxicity-assay was normalized to vehicle (1% DMSO). Values are expressed as mean ± SEM of three triplicate experiments, * p<0.05, ** p<0.01, *** p<0.001. (C) mRNA expression levels of FXR target genes (*Fgf19, Ibabp*) in human colon cancer cells after treatment with 100 µM isoBAs in the presence or absence of FXR agonists (2 µM GW4064, 10 µM OCA); n=3. Values represent the mean ± SEM. **p<0.01, ***p<0.001.

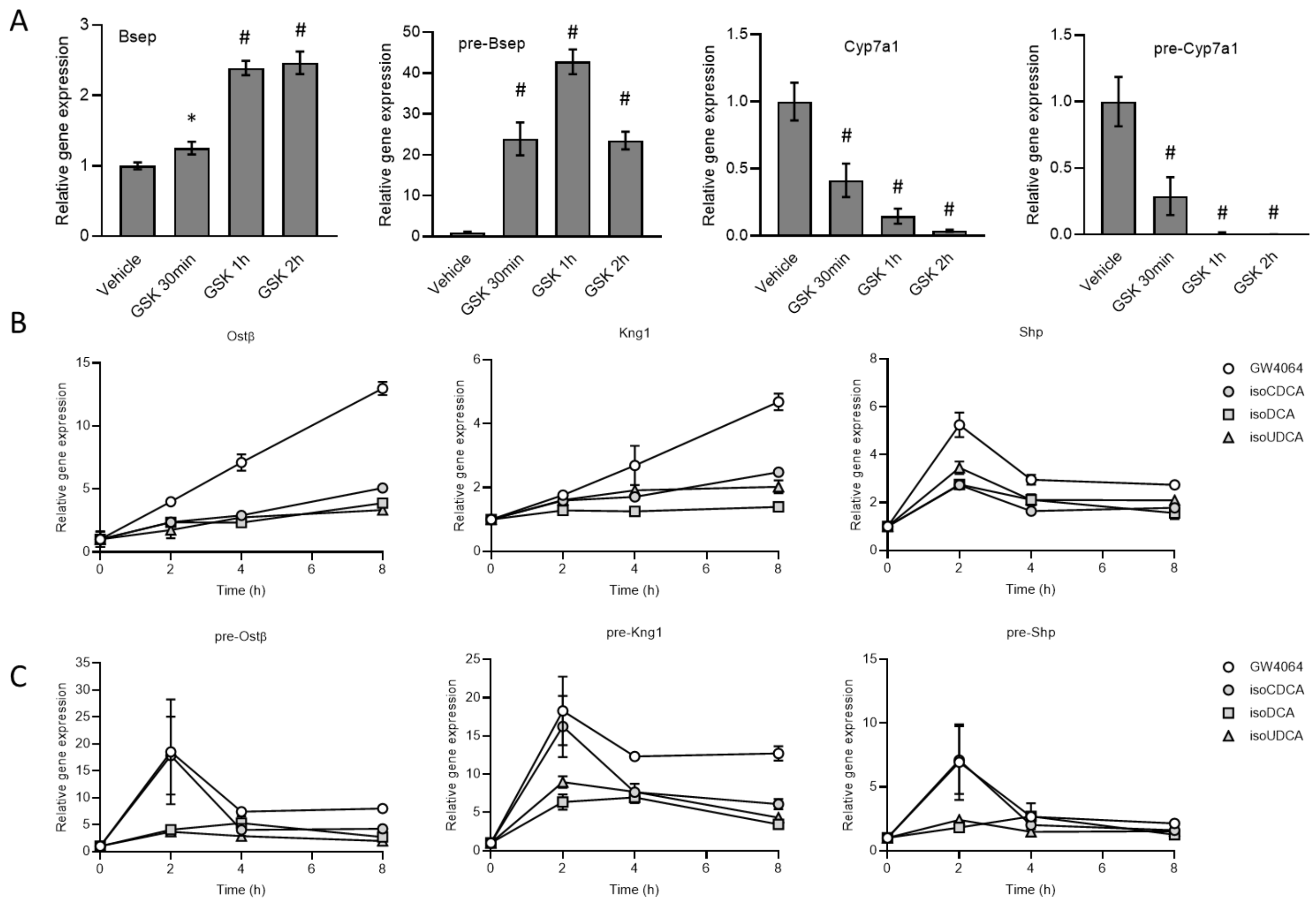

Supplementary Figure 2. (A) Hepatic expression of bile acid transport (Bsep) and BA-synthesis gene (Cyp7a1) pre-mRNA and mRNA after treatment with either vehicle or the synthetic FXR agonist GSK2324 for 30 minutes (n = 7-8), 1 hour (n = 8), or 2 hours (n = 7-8) before sacrifice; values represent the mean ± SEM. *p<0.05, #p<0.01. Effect of the synthetic FXR ligand (GW4064) and isoBAs (isoCDCA, isoDCA, isoUDCA) on mRNA (B) and pre-mRNA (C) levels of *Ost*b, *Kng*1, *Shp* in HepG2 cells treated over time; n=3. Values represent the mean ± SEM.

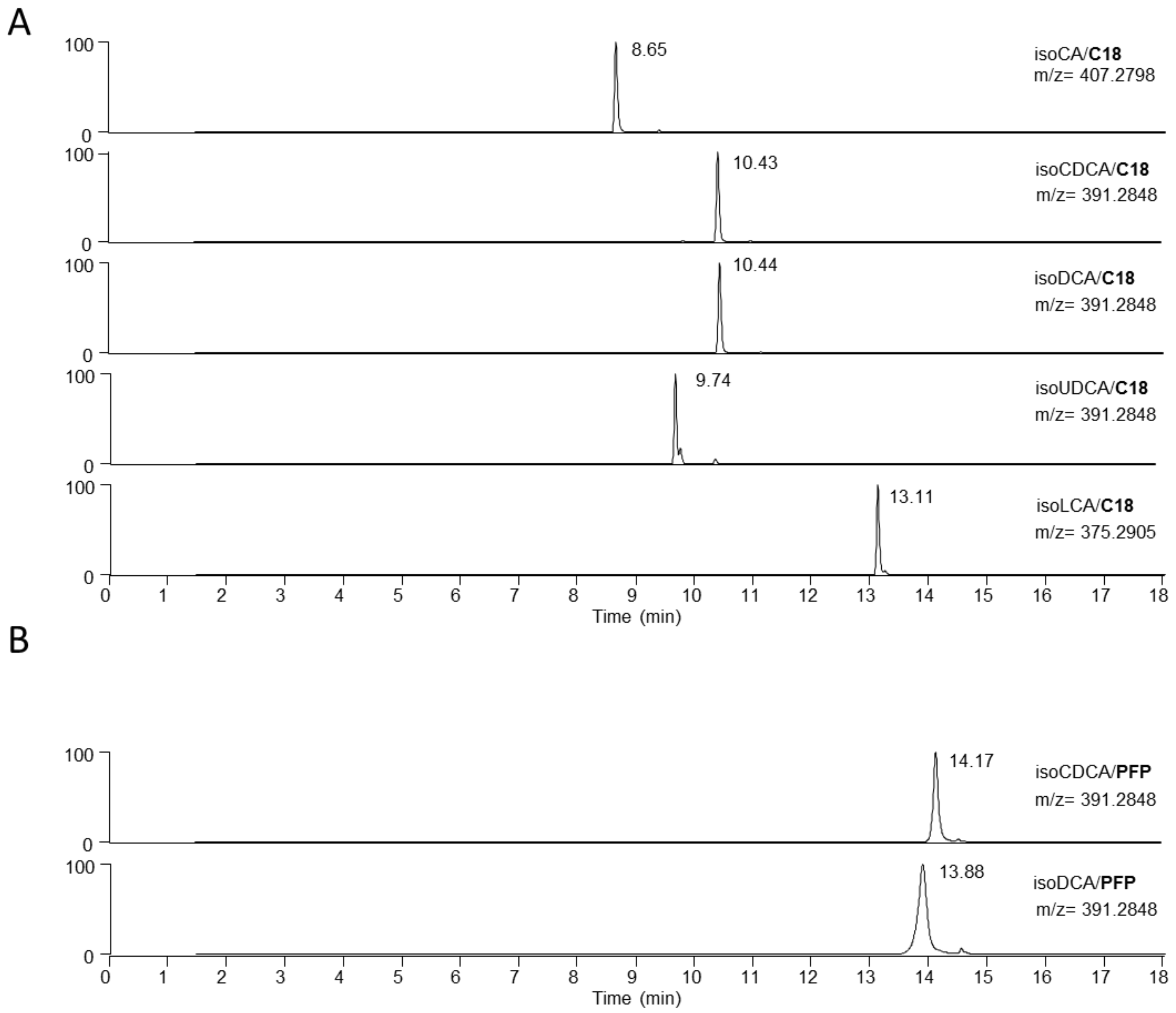

Supplementary Figure 3. Liquid chromatography–mass spectrometry (LC-MS) of synthesized isoBAs on (A) C-18 and (B) Pentafluorophenyl propyl (PFP) columns. 3b, 7a-dihydroxy and 3b, 12a-dihydroxy (isoCDCA and isoDCA) have identical retention times on the C18 column. LC/MS chromatography using PFP column allows separation of isoCDCA and isoDCA. (m/z) mass divided by charge number.

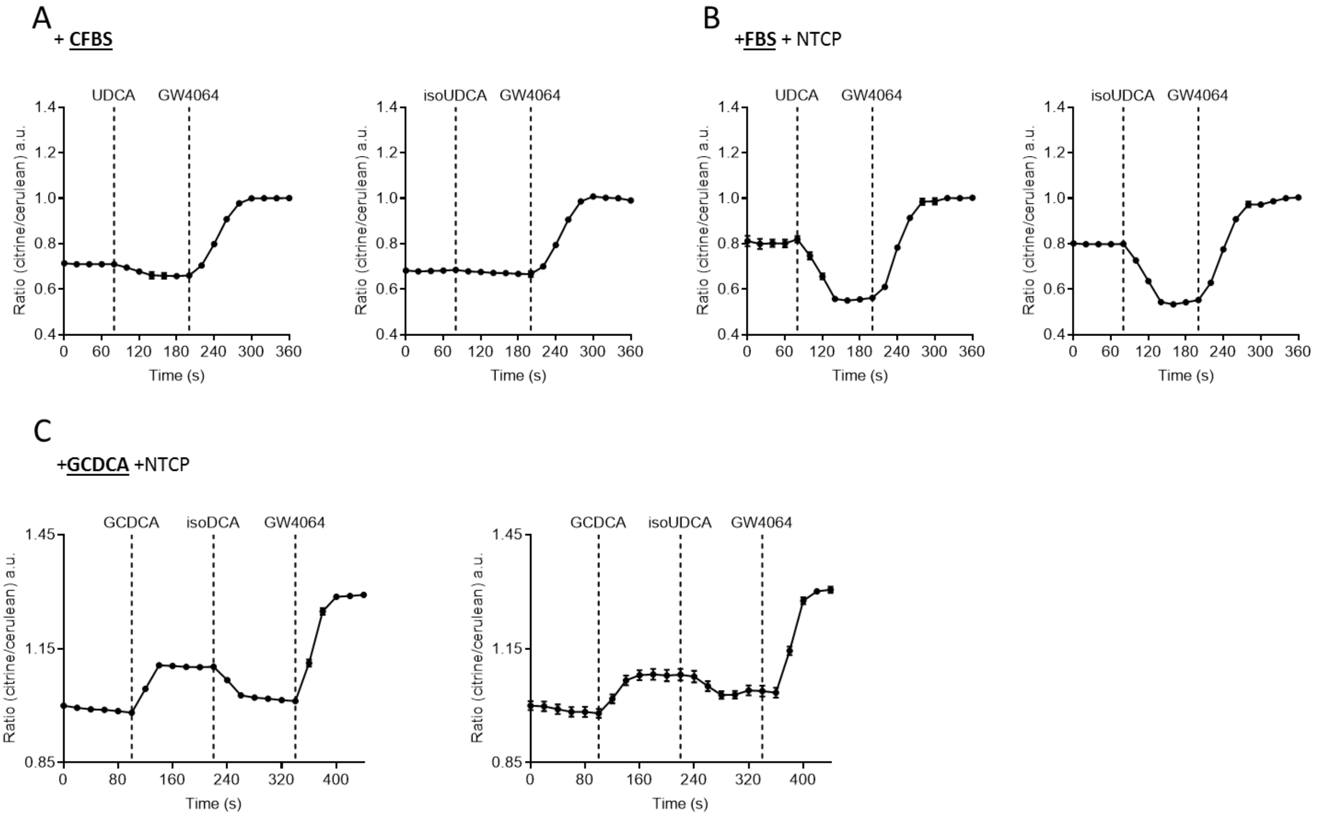

Supplementary Figure 4. Changes in FRET emission ratio upon addition of (A) 100µM UDCA or isoUDCA in **CFBS** and (B) 100µM UDCA or isoUDCA in **FBS** in nucleoBAS-NTCP-mKate2 expressing cells and 5µM GW4064 at t=200s. (C) FRET emission ratios in nucleoBAS-NTCP-mKate2 expressing U2OS cells treated with glycine conjugated CDCA (GCDCA) in DMEM containing charcoal treated serum (CFBS). 10µM GCDCA, 100µM isoDCA or isoUDCA and 5µM GW4064 were added at time points indicated. n=6 cells per experiment; Error bars represent the SEM.

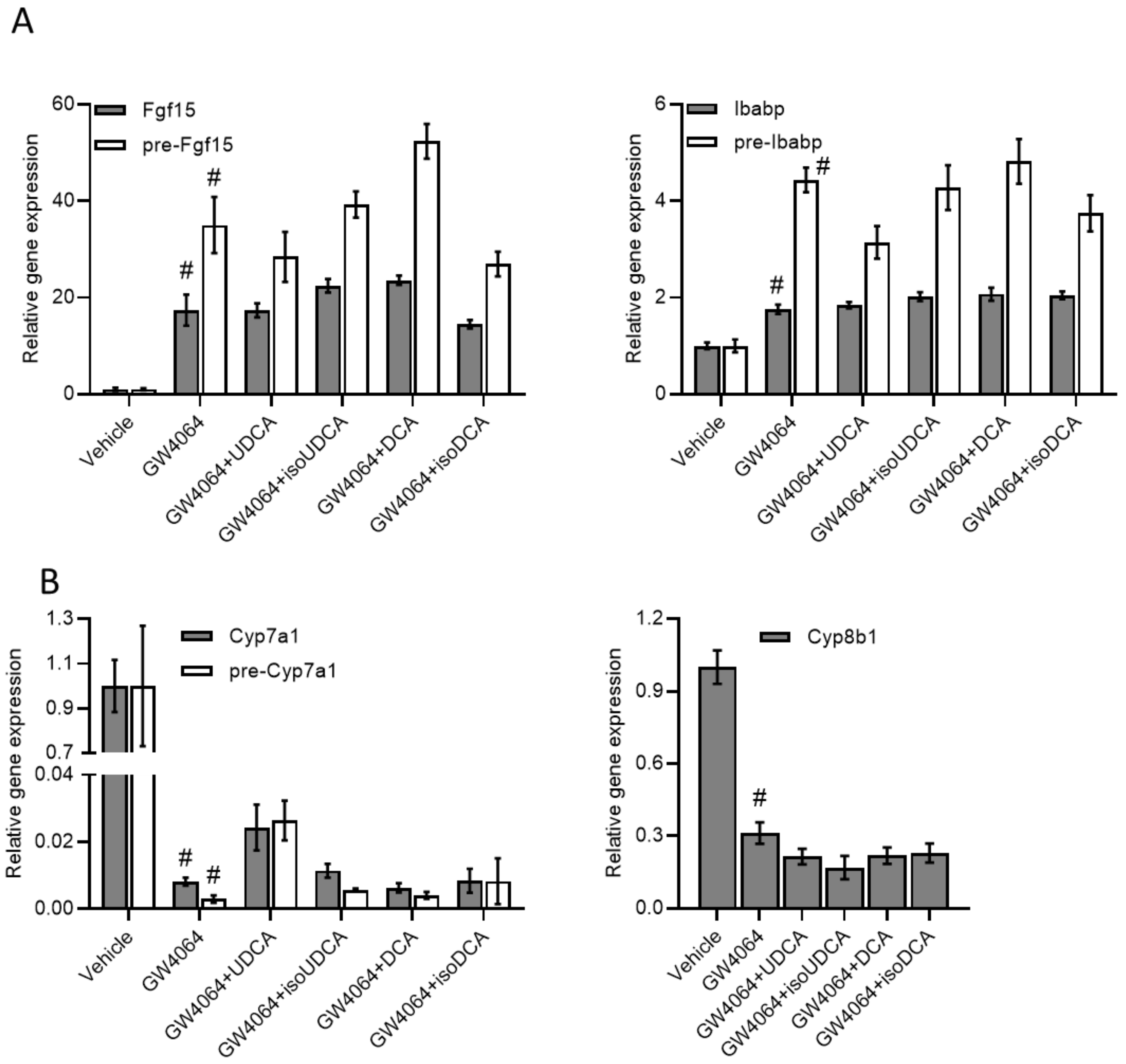

Supplementary Figure 5. Regulation of FXR target genes *in vivo* by single dose administration of secondary 3α-hydroxy BAs or isoBAs in combination with GW4064. Supplement to Figure 5. (A) Regulation of Fgf15 and Ibabp pre-mRNA and mRNA by GW4046, UDCA+GW4064, isoUDCA+GW4064, DCA+GW4064 and isoDCA+GW4064. (B) Repression of bile acids synthesis genes (Cyp7a1 and Cyp8b1) in livers of mice treated with 3α-hydroxy BAs or isoBAs and GW4064. Administration of BAs followed by GW4064 with 1h time-delay for a total of 6h. Mice were gavaged with olive oil (vehicle), GW4064 (30mg/kg BW), and BAs (60mg/kg BW). Genes were analyzed 6 hours after gavage; n=3-4, values represent the mean ± SEM. *p<0.05; #p<0.01.

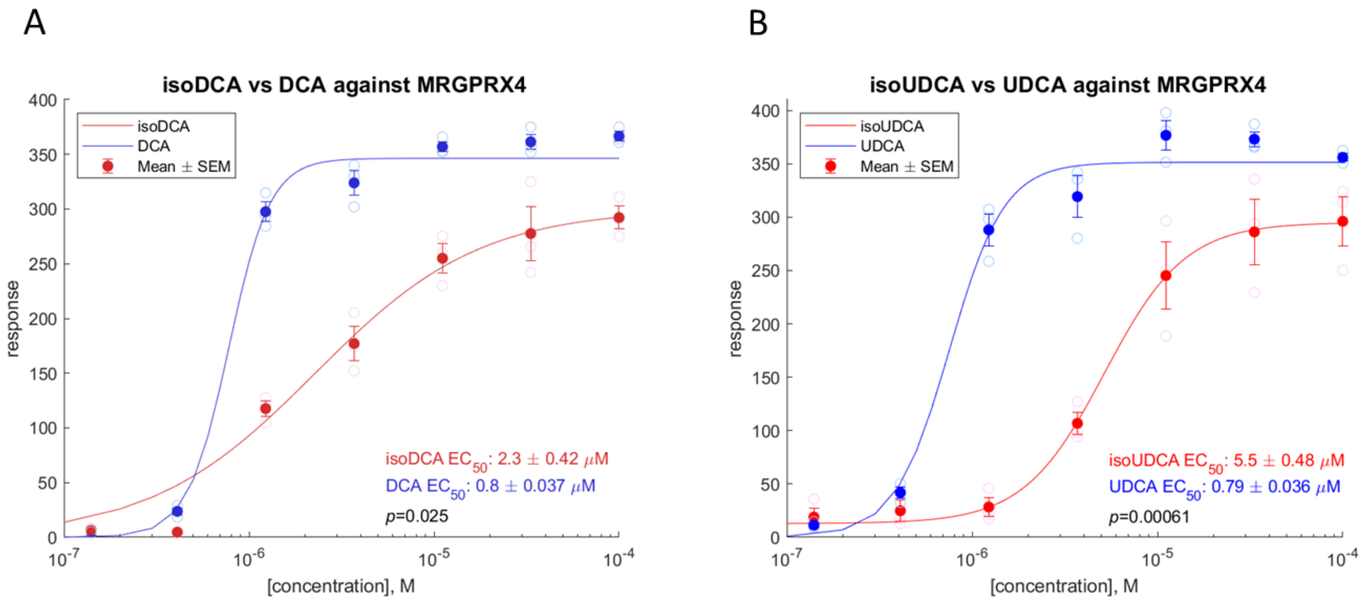

Supplementary Figure 6. Activation of MRGPRX4 by secondary isoBAs and 3alpha-epimers. Dose dependent Ca2+ response curves to different BAs in MRGPRX4-expressing HEK293 cells; (A) isoDCA and DCA, (B) isoUDCA and UDCA. Data are a representative experiment of three independent experiments performed in triplicate; values represent the mean ± SEM.

IsoBA synthesis and BA-standards used in this study

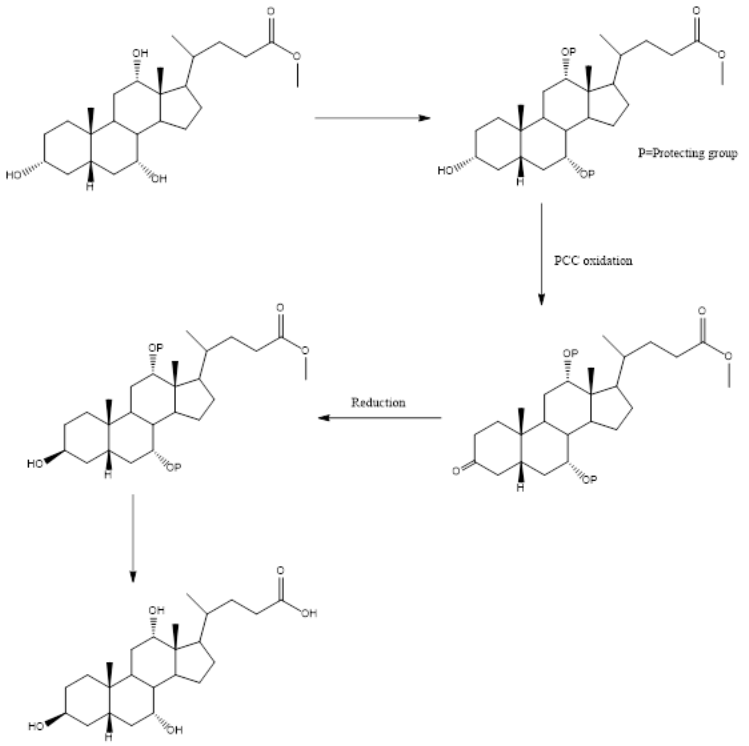

Scheme 1 (Cholic acid is taken as a typical example to show the general strategic approach)

All chemicals used were purchased from Sigma-Aldrich (Dublin, Ireland), excepted where stated. All chemical reactions were monitored by TLC. Uncorrected melting points were measured using a Stuart SMP11 melting point apparatus and were uncorrected. IR spectra were acquired on a Perkin Elmer 205 FT infrared Paragon 1000 spectrometer, with wavenumber given in unit cm^{-1}. Solid samples were presented using KBr disk and oils were measured as neat films on NaCl plates. ^{1}H and ^{13}C nuclear magnetic resonance (NMR) spectra were measured at the temperature of 27ºC on a Bruker DPX 400 and an Agilent 40MR DD2 spectrometer (400.13MHz, ^{1}H; 100.61MHz, ^{13}C) using tetramethylsilane (TMS) as internal standard, in $CDCl_3$. Coupling constants were measured in Hz. For ^{1}H-NMR, chemical shifts were reported: shift value (number of protons, multiplicity of the peak, coupling constants where applicable). Electrospray ionization mass spectrometry (ESI-MS) was performed in the positive ion mode on a liquid chromatography time-of-flight mass spectrometer (Micromass LCT, waters Ltd., Manchester, UK). Compound purity/accuracy was confirmed using a combination of ^{1}H-NMR, ^{13}C-NMR, TLC and HR-MS.

General procedure for the formation of 3β-OH bile acids with L-selectride solution

To a cooled solution of the 24-methyl ester protected bile acid 3-ketone (0.90 g, 2.015 mmol) in dry THF (15 ml) was dropwise added a solution of L-selectride (1.8 ml, 4 eq.) under N_2 condition and the mixture was stirred at -18°C for over one hour and then allowed to warm to RT and stirred for overnight. TLC analysis showed formation of two closely separated products (mobile phase hexane: EtOAc=1:1). The mixture was quenched with a saturated ammonium chloride solution (100 ml) and then extracted with DCM (3×50 ml). The combined organic layer was washed with brine (100 ml) and was dried over $MgSO_4$ and filtered. The solvent was removed in vacuum and then purified by flash column chromatography (hexane: EtOAc=1:1) to yield majority of beta product (0.82 g, 90.35%) and a small amount of alpha hydroxy compound (0.09 g, 9.65%).

3β-hydroxy-12α-hydroxy-5β-cholanoate (3β-OH DCA)

^{1}H-NMR δ ($CDCl_3$): 0.67 (3H, s, 18-CH_3), 0.93 (3H, s, 19-CH_3), 0.95 (3H, d, J = 6.2 Hz, 21-CH_3), 1.04-1.16 (4H, m, 8-CH, 9-CH, 14-CH, 17-CH), 1.23-1.33 (3H, m, 1-CH_2, 20-CH), 1.35-1.60 (10H, m, 2-CH_2, 4-CH_2, 6-CH_2, 7-CH_2, 11-CH_2), 1.61-1.76 (4H, m, 15-CH_2, 16-CH_2), 1.77-1.85 (2H, m, 22-CH_2), 2.22-2.38 (2H, m, 23-CH_2), 3.98 (1H,s, 12β-H), 4.09 (1H, s, 3α-H). ^{13}C-NMR ppm ($CDCl_3$): 12.7 (18-C, CH_3), 17.3 (19-C, CH_3), 23.6 (21-C, CH_3), 25.9 (15-C, CH_2), 26.5 (16-C, CH_2), 27.4 (6-C, CH_2), 28.9 (11-C, CH_2), 30.7 (2-C, CH_2), 30.8 (22-C, CH_2), 32.8 (23-C, CH_2), 32.9 (7-C, CH_2), 35.0 (20-C, CH), 35.8 (1-C, CH_2), 35.9 (8-C, 9-C, CH), 36.3 (4-C, CH_2), 36.4 (5C-CH), 46.5 (13-C, <u>C</u>-CH_3), 47.3(17-C, CH), 48.3 (14-C, CH), 67.2 (3-C, CH), 73.3 (12-C, CH), 179.3 (24-C, COOH). HRMS: Found: $(M+H)^+$ = 392.2852, calculated $C_{24}H_{39}O_4^+$=392.2854.

3β-hydroxy-7α-hydroxy-5β-cholanoate (3β-OH CDCA)

^{1}H-NMR δ ($CDCl_3$): 0.64 (3H, s, 18-CH_3), 0.93 (6H, m, 19-CH_3, 21-CH_3), 1.04-1.15 (4H, m, 8-CH, 9-CH, 14-CH, 17-CH), 1.21-1.32 (3H, m, 1-CH_2, 20-CH), 1.36-1.60 (10H, m, 2-CH_2, 4-CH_2, 6-CH_2, 7-CH_2, 11-CH_2), 1.61-1.74 (4H, m, 15-CH_2, 16-CH_2), 1.75-1.86 (2H, m, 22-CH_2), 2.20-2.43 (2H, m, 23-CH_2), 3.85 (1H, d, 7β-H), 4.06 (1H, s, 3α-H). ^{13}C-NMR ppm ($CDCl_3$): 11.8 (18-C, CH_3), 18.3 (19-C, CH_3), 21.0 (21-C, CH_3), 21.6 (11-C, CH_2), 23.2 (15-C, CH_2), 28.0 (16-C, CH_2), 29.8 (2-C, CH_2), 30.8 (22-C, CH_2), 31.1 (23-C, CH_2), 35.7 (1-C, CH_2), 35.8 (20-C, CH), 36.0 (10-C, <u>C</u>-CH_3), 37.3 (4-C, CH_2), 39.0 (6-C, CH) 39.3 (8-C, CH), 39.7 (12-C, CH_2), 41.9 (5-C, CH), 42.8 (13-C, <u>C</u>-CH_3), 50.5 (14-C, CH), 55.7 (17-C, CH), 68.8 (7-C, CH), 71.7 (3-C, CH), 178.6 (24-C, COOH). HRMS: Found: $(M+H)^+$ = 392.2847, calculated $C_{24}H_{39}O_4^+$=392.2854.

3β-hydroxy-5β-cholanoate (3β-OH LCA)

^{1}H-NMR δ ($CDCl_3$): 0.63 (3H, s, 18-CH_3), 0.90 (3H, d, J = 6.1 Hz, 21-CH_3), 0.94 (3H, s, 19-CH_3), 1.00-1.16 (4H, m, 8-CH, 9-CH, 14-CH, 17-CH), 1.24-1.32 (3H, m, 1-CH_2, 20-CH), 1.38-1.56 (10H, m, 2-CH_2, 4-CH_2, 6-CH_2, 7-CH_2, 11-CH_2), 1.67-1.78 (4H, m, 15-CH_2, 16-CH_2), 1.80-1.88 (2H, m, 22-CH_2), 2.22-2.40 (2H, m, 23-CH_2), 4.09 (1H, s, 3α-H). ^{13}C-NMR ppm ($CDCl_3$): 12.1 (18-C, CH_3), 18.2 (19-C, CH_3), 21.1 (21-C, CH_3), 24.2 (11-C, CH_2), 26.5 (15-C, CH_2), 27.4 (16-C, CH_2), 28.2 (6-C, CH_2), 30.7 (2-C, CH_2), 30.8 (22-C, CH_2), 32.8 (7-C, CH_2), 32.8 (23-C, CH_2), 32.9 (7-C, CH_2), 34.6 (20-C, CH), 35.1 (10-C, C-CH_3), 35.8 (1-C, CH_2), 35.9 (8-C, CH), 36.5 (4-C, CH_2), 46.5 (5-C, CH), 47.3 (12-C, CH_2), 48.3 (C-CH_3), 55.0 (9-C, CH), 55.9 (17-C, CH), 56.7 (14-C, CH), 67.2 (3-C, CH), 179.3 (24-C, COOH). HRMS: Found: $(M+H)^+$ =376.2902, calculated $C_{24}H_{39}O_3^+$=376.2905.

3β-hydroxy-7α-hydroxy-12α-hydroxyl-5β-cholanoate (3β-OH CA)

^{1}H-NMR δ ($CDCl_3$): 0.69 (3H, s, 18-CH_3), 0.93 (3H, s, 19-CH_3), 0.98 (3H, d, J = 6.2 Hz, 21-CH_3), 1.13-1.24 (4H, m, 8-CH, 9-CH, 14-CH, 17-CH), 1.24-1.40 (3H, m, 1-CH_2, 20-CH), 1.40-1.56 (10H, m, 2-CH_2, 4-CH_2, 6-CH_2, 7-CH_2, 11-CH_2), 1.72-1.77 (4H, m, 15-CH_2, 16-CH_2), 1.80-1.88 (2H, m, 22-CH_2), 2.37-2.47 (2H, m, 23-CH_2), 3.85 (1H, d, 7β-H), 3.98 (1H, s, 12β-H), 4.06 (1H, s, 3α-H). ^{13}C-NMR ppm ($CDCl_3$): 12.1 (18-CH_3), 14.2 (19-CH_3), 18.4 (21-CH_3), 21.0 (15-C, CH_2), 23.5 (16-C, CH_2), 26.9 (9-C, CH), 28.6 (11-C, CH_2), 30.8 (2-C, CH_2), 30.9 (22-C, CH), 34.1 (23-C, CH_2), 34.2 (1-C, CH_2), 35.2 (20-C, CH), 37.2 (4-C & 6-C, CH_2), 39.4 (10-C, C-CH_3), 40.2 (8-C, CH), 43.8 (5-C, C-CH_3), 49.8 (14-C, CH), 55.0 (17-C,CH), 68.8 (7-C, CH), 71.4 (3-C, CH), 73.3 (12-C, CH), 179.2 (24-C, COOH). HRMS: Found: $(M+H)^+$ =408.2802, calculated $C_{24}H_{39}O_5^+$ =408.2803.

3β-hydroxy-7β-hydroxy-5β-cholanoate (3β-OH UDCA)

^{1}H-NMR δ ($CDCl_3$): 0.67 (3H, s, 18-CH_3), 0.93 (3H, d, J = 6.1 Hz, 21-CH_3),

0.97 (3H, s, 19-CH_3), 1.04-1.20 (4H, m, 8-CH, 9-CH, 14-CH, 17-CH), 1.24-1.43 (3H, m, 1-CH_2, 20-CH), 1.43-1.52 (10H, m, 2-CH_2, 4-CH_2, 6-CH_2, 7-CH_2, 11-CH_2), 1.74-1.78 (4H, m, 15-CH_2, 16-CH_2), 1.82-1.83 (2H, m, 22-CH_2), 2.35-2.39 (2H, m, 23-CH_2), 3.54 (1H, m, 7α-H), 4.06 (1H, s, 3α-H). ^{13}C-NMR ppm ($CDCl_3$): 12.2 (19-C, CH_3), 18.4 (18-C, CH_3), 21.5 (21-C, CH_3), 23.9 (11-C, CH_2), 26.9 (5-C, CH_2), 28.6 (6-C, CH_2), 30.6 (2-C, CH_2), 30.9 (22-C, CH_2), 34.5 (23-C, CH_2), 35.1 (20-C, CH), 35.2 (10-C, CH_2), 36.9 (1-C, CH_2), 37.2 (4-C & 6-C, CH_2), 38.6 (8-C, CH), 40.1 (5-C, CH), 40.2 (9-C, CH), 43.6 (12-C, CH_2), 43.8 (13-C, C-CH_3), 55.0 (14-C, CH), 55.9 (17-C, CH). 66.7 (7-C, CH), 71.5 (3-C, CH), 177.8 (24-C, COOH). HRMS: Found: $(M+H)^+$ =392.2848, calculated $C_{24}H_{39}O_4^+$=392.2854.

1. Bile acids and keto/oxo intermediates used as standards for LC/MS/MS.

Common Name	Abbreviation	**Name**	**CAS No.:**	**Mass (-1)**
7-Keto deoxycholic acid	7KDC	3alpha,12alpha-dihydroxy-7-oxo-5β-cholanic acid	CAS 911-40-0	405,2641
7-Keto lithocholic acid	7KL	3-alpha-Hydroxy-7-oxo-5-beta-cholan-24-oic acid	CAS 4651-67-6	389,2692
3-oxo deoxycholic acid	3oxoDC	5-β-CHOLANIC ACID-12-ALPHA-DIOL-3-ONE	CAS 07-01-4185	389,2692
(3β) Iso-deoxycholic acid	**IDC**	5β-CHOLANIC ACID-3β, 12α-DIOL	CAS 570-63-8	391,2848
3-keto deoxycholic acid = 3-DEHYDROCHOLIC ACID	3KDC	5-β-CHOLANIC ACID-7-α 12-α-DIOL-3-ONE	CAS 2304-89-4	405,2641
Ursocholic acid = 7-Epicholic acid	UC	3α,7β,12α-Trihydroxy-5beta-cholan-24-oic acid	CAS 2955-27-3	407,2797
12β-hydroxy isocholic acid = lago deoxycholic acid	12βIC	5β-CHOLANIC ACID-3α, 7α, 12β-TRIOL	CAS 71883-64-2	407,2797
12-Ketolithocholic acid = 12-Ketodeoxycholic acid	12KL	5-β-CHOLANIC ACID-3-α-OL-12-ONE	CAS 5130-29-0	389,2692
Iso-deoxycholic acid	**IDC/wrong**	5β-CHOLANIC ACID-7α, 12α-DIOL	CAS 566-17-6	391,2848
https://www.steraloids.com/5cholanic-acid-1/chol-based/5-cholanic-acid-7-12-diol.html				
**isoBAs synthesized and used in this study**				
(3β) Iso-chenodeoxycholic acid	isoCDCA	5β-CHOLANIC ACID-3β, 7α-DIOL		391,2848
(3β) Iso-cholic acid	isoCA	5β-CHOLANIC ACID-3β, 7α, 12α-TRIOL		407,2797
(3β) Iso-deoxycholic acid	isoDCA	5β-CHOLANIC ACID-3β, 12α-DIOL		391,2848
(3β) Iso-ursodeoxycholic acid	isoUDCA	5β-CHOLANIC ACID-3β, 7β-DIOL		391,2848
(3β) Iso-lithocholic acid	isoLCA	5β-CHOLANIC ACID-3β-OL		391,2848
**Conjugated and unconjugated primary and secondary BAs were purchased from:**	TRC -canada	Steraloids Inc.	Sigma	
